# Summary of Natural Products Ameliorate Concanavalin A-Induced Liver Injury: Structures, Sources, Pharmacological Effects, and Mechanisms of Action

**DOI:** 10.3390/plants10020228

**Published:** 2021-01-25

**Authors:** Sabrin R. M. Ibrahim, Alaa Sirwi, Basma G. Eid, Shaimaa G. A. Mohamed, Gamal A. Mohamed

**Affiliations:** 1 Department of Pharmacognosy, Faculty of Pharmacy, Assiut University, Assiut 71526, Egypt; 2 Department of Natural Products and Alternative Medicine, Faculty of Pharmacy, King Abdulaziz University, Jeddah 21589, Saudi Arabia; asirwi@kau.edu.sa (A.S.); gahussein@kau.edu.sa (G.A.M.); 3 Department of Pharmacology and Toxicology, Faculty of Pharmacy, King Abdulaziz University, Jeddah 21589, Saudi Arabia; beid@kau.edu.sa; 4 Faculty of Dentistry, British University, El Sherouk City, Suez Desert Road, Cairo 11837, Egypt; shaimaag1973@gmail.com; 5 Department of Pharmacognosy, Faculty of Pharmacy, Al-Azhar University, Assiut Branch, Assiut 71524, Egypt

**Keywords:** liver diseases, autoimmune hepatitis, inflammation, concanavalin A, natural products, drug discovery, mechanism of action

## Abstract

Liver diseases represent a threat to human health and are a significant cause of mortality and morbidity worldwide. Autoimmune hepatitis (AIH) is a progressive and chronic hepatic inflammatory disease, which may lead to severe complications. Concanavalin A (Con A)-induced hepatic injury is regarded as an appropriate experimental model for investigating the pathology and mechanisms involved in liver injury mediated by immune cells as well as T cell-related liver disease. Despite the advances in modern medicine, the only available strategies to treat AIH, include the use of steroids either solely or with immunosuppressant drugs. Unfortunately, this currently available treatment is associated with significant side-effects. Therefore, there is an urgent need for safe and effective drugs to replace and/or supplement those in current use. Natural products have been utilized for treating liver disorders and have become a promising therapy for various liver disorders. In this review, the natural compounds and herbal formulations as well as extracts and/or fractions with protection against liver injury caused by Con A and the underlying possible mechanism(s) of action are reviewed. A total of 53 compounds from different structural classes are discussed and over 97 references are cited. The goal of this review is to attract the interest of pharmacologists, natural product researchers, and synthetic chemists for discovering novel drug candidates for treating immune-mediated liver injury.

## 1. Introduction

**Figure 1:**
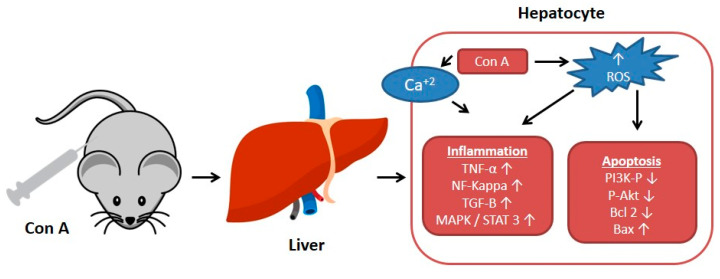
Effect of concanavalin A (Con A) on the molecular level in Con A-induced hepatotoxicity. NF-kB: Nuclear factor-kappa B; ROS: Reactive oxygen species; TGF-B: Transforming growth factor B; MAPK: Mitogen-activated protein kinase; Stat3: Signal transducer and activator of transcription 3; p-AKT: Phospho-protein kinase B; PI3K: Phosphatidylinositol 3 kinase; TNF-α: Tumor necrosis factor α.

The plant lectin, concanavalin A (Con A) separated from *Canavalia ensiformis* (jack bean) is known as a T-lymphocytes activator. T-lymphocytes are effector cells, which play a remarkable role in the immuno-stimulatory process in case of allograft rejection, viral infection, or autoimmune diseases in mammals [1]. Con A stimulates T-cell causing a release of several cytokines such as tumor necrosis factor-α (TNF-α), interferon-gamma (IFN-γ)*,* granulocyte macrophage-colony stimulating factor (GM-CSF), and interleukins (ILs), that maintain inflammatory and immuno-stimulatory processes and may arouse acute toxicity [2]. Therefore, Con A-activation of T-cell leads to cytokine-induced hepatic injury, which can be assessed by electron microscopy of the liver and by determining the plasma levels of transaminases [1,3]. This injury is characterized by severe liver inflammation and massive hepatocyte apoptosis/necrosis [4,5,6]. Con A produces oxidative stress by increasing the ROS levels and decreasing antioxidants levels (e.g., glutathione, SH), which leads to an increase in intracellular Ca^+2^ and accelerates lipid peroxidation that damages the cell membrane and other cellular components [7,8]. Also, Con A induces inflammation in the hepatic tissue through elevating the levels of TNF-α, adhesion molecules, transforming growth factor-β1 (TGF-β1), mitogen-activated protein kinases (MAPKs), and signal transducer and activator of transcription 3 (STAT3) [9,10,11]. Moreover, apoptosis can be induced by Con A through suppressing Akt (p-Akt), phosphatidylinositol 3 kinase (PI3K), and Bcl2 and upregulating Bax [12,13,14,15] (Figure 1).

It was reported that Con A-immune responses depend on various cells such as natural killer T (NKT) cells, CD4^+^ T cells, neutrophils, and intrahepatic macrophages namely, Kupffer cells (KCs) [16]. Con A has been utilized as an insulin receptors agonist and in general T-cell biology [17,18]. This is a common model for immune-related hepatitis [19]. The pathogenesis in this model is unique and it offers several similarities to acute liver diseases seen in human beings such as: acute liver failure, autoimmune hepatitis (AIH), and acute viral hepatitis in which T-cells involvement and immune activation/infiltration were observed. This model selectively details the T-cell functions in inflammatory liver disease [3,6,19]. Therefore, the Con A model is utilized to study the pathogenesis, microscopic morphological changes, and effects of potential treatments for AIH and is recognized as an acceptable and well-characterized model for liver injury mediated by immune responses [20,21].

The liver is a vital organ in nutrients metabolism, immune surveillance, and toxin clearance. However, it can be destroyed by the overactive immune responses that can be triggered by certain medications, metabolic diseases, and toxins, as well as intravascular infections [22]. Liver diseases, including viral and autoimmune hepatitis as well as drug-induced liver damage, are a major culprit of mortality and morbidity worldwide and represent a threat to human well-being [4].

Autoimmune hepatitis (AIH) is a hepatic inflammatory condition, in which the liver parenchyma is destroyed, with lymphocyte infiltration, necrosis, and apoptosis of liver cells, and a rise in the level of transaminase enzymes [21,23,24]. AIH may lead to an array of debilitating complications, including encephalopathy, ascites, cirrhosis, hepatocellular carcinoma, and/or death [20]. The current available management options for AIH include administering steroids (e.g., prednisone, prednisolone) on their own or combining them with immunosuppressive drugs (e.g., azathioprine) [9,25]. However, these medications are non-specific and have significant adverse effects that prompt many patients to discontinue their use [25,26]. Thus, there is an urgent need for discovering more safer and specific therapeutic agents.

**Figure 2:**
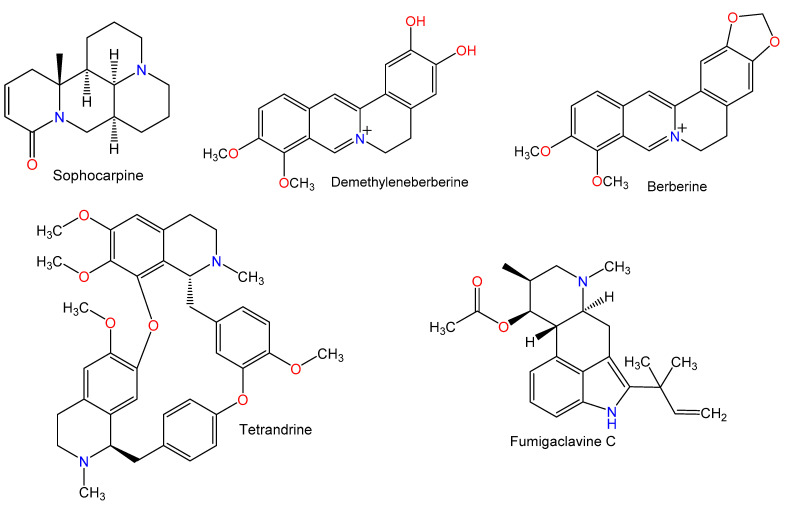

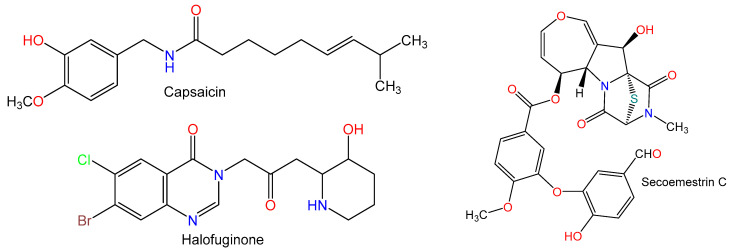
Chemical structures of alkaloids.

**Figure 3:**
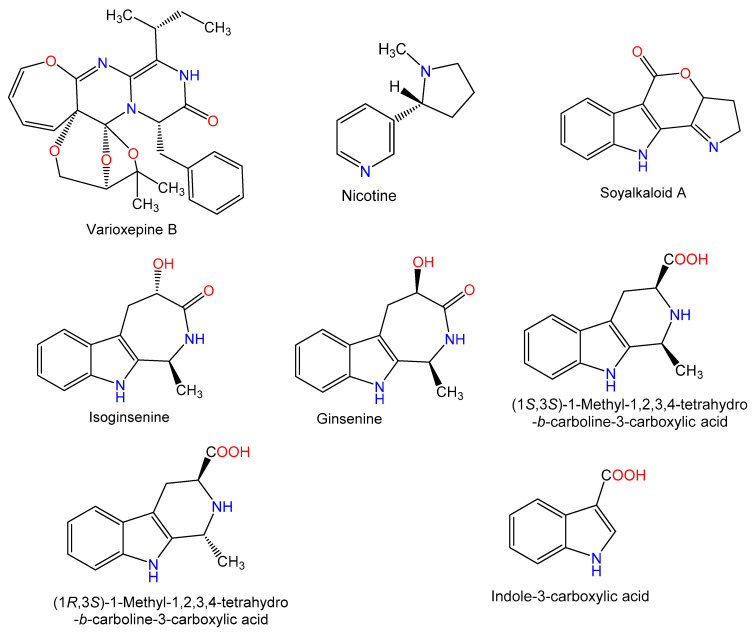
Chemical structures of alkaloids.

**Figure 4:**
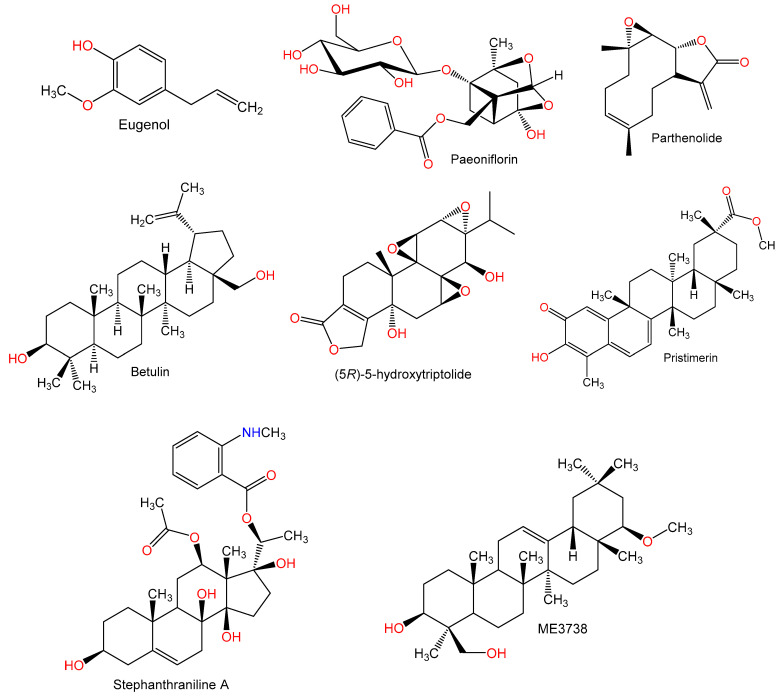
Chemical structures of terpenes.

**Figure 5:**
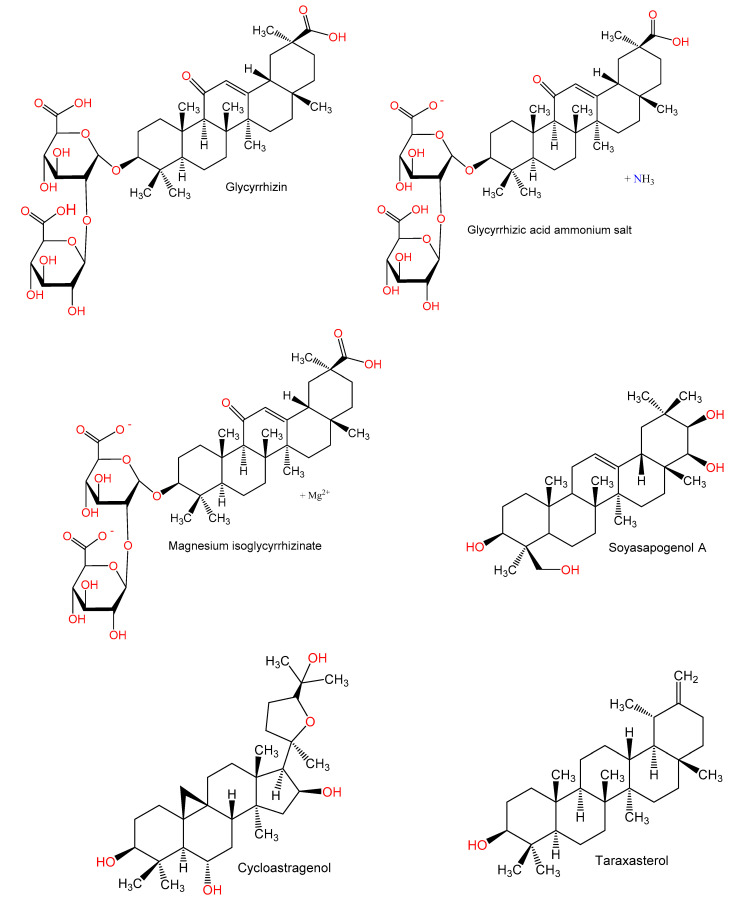
Chemical structures of terpenes.

**Figure 6:**
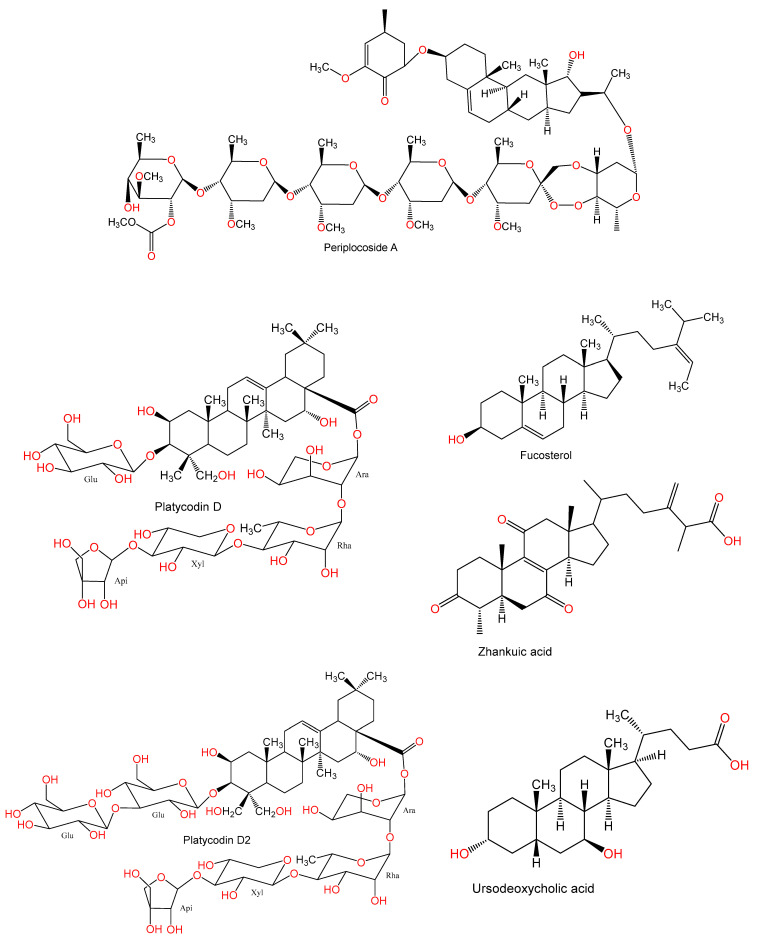
Chemical structures of terpenes and sterols.

**Figure 7:**
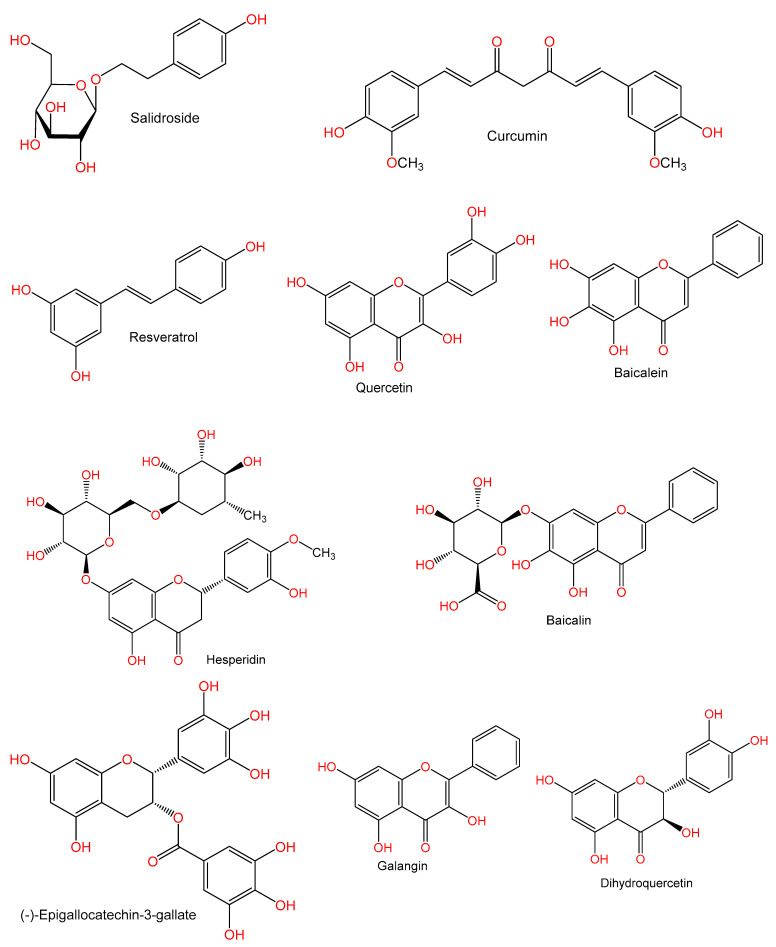
Chemical structures of phenolics.

**Figure 8:**
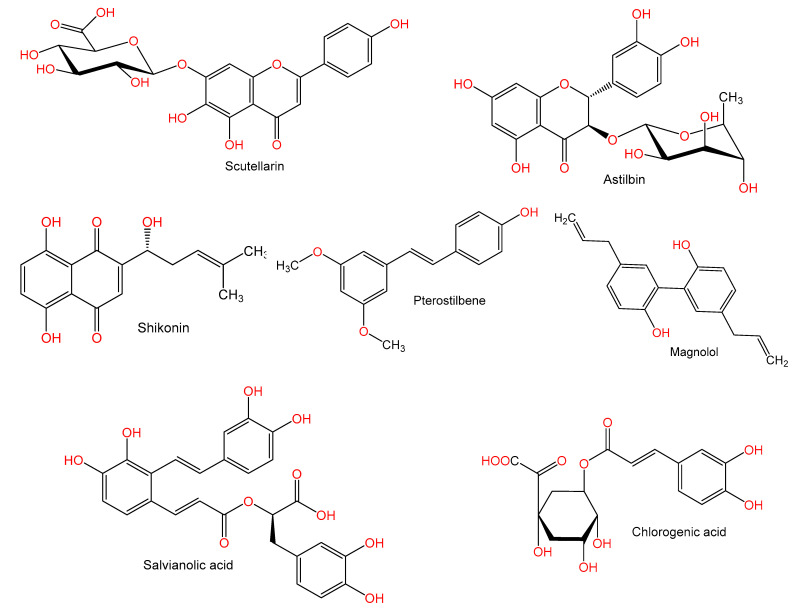
Chemical structures of phenolics.

**Figure 9:**
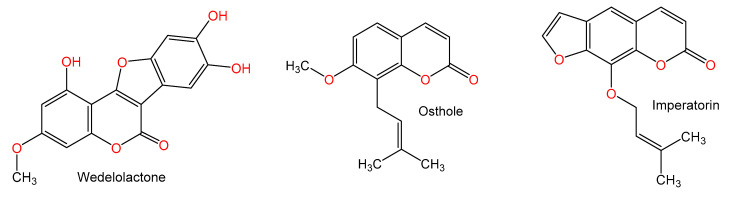

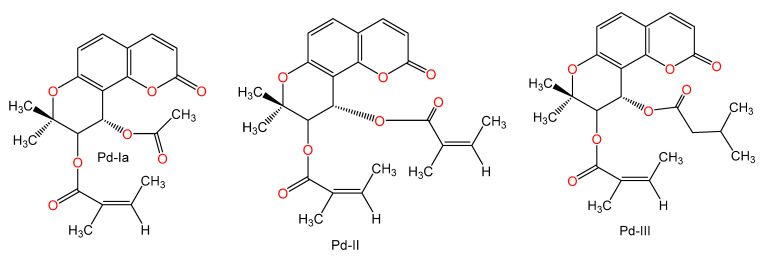
Chemical structures of coumarin and coumarin derivatives.

**Figure 10:**
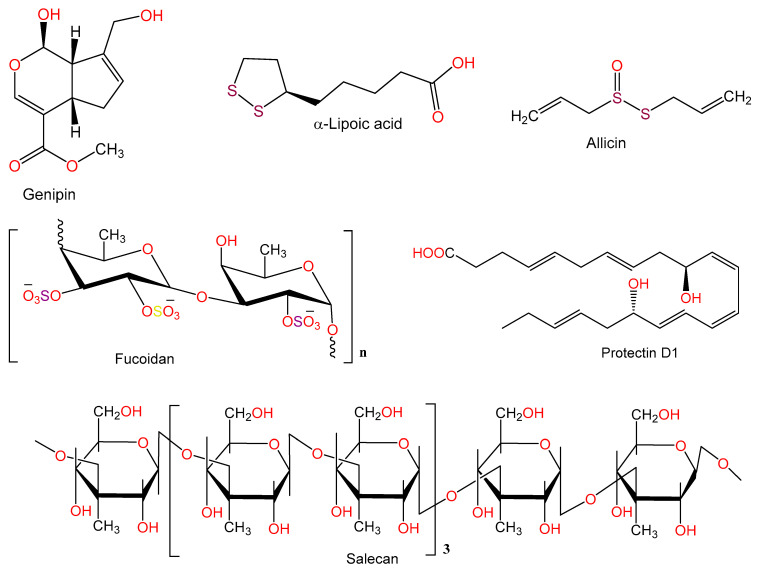
Chemical structures of other compounds.

For hundreds of years, herbal medicines have been utilized for treating liver disorders and have become a promising therapy for various liver disorders [27,28]. Many of the therapeutic agents used in liver diseases are either natural products or natural product derivatives because of their capability to act on various biological targets [28,29,30]. Moreover, there is a recent expansion of interest in the discovery of natural products from different sources—e.g., terrestrial plants, marine organisms, and microorganisms—as potential leads for treating AIH. In this review, the natural compounds and herbal formulation as well as extracts and/or fractions reported to ameliorate Con A-induced hepatitis and their mechanism of action are reviewed (Figure 2, Figure 3, Figure 4, Figure 5, Figure 6, Figure 7, Figure 8, Figure 9 and Figure 10; Table 1, Table 2 and Table 3). This review was performed using the databases; Google Scholar, Science Direct, Springer Link, JACS, Taylor & Francis, Web of Science, Scopus, Bentham Science, or Wiley Online Library. The isolated compounds from different natural sources are classified into different chemical groups. Moreover, their sources, structures, molecular weights, and formulae as well as the effects and possible mechanisms are highlighted (Table 1 and Table 2; Figure 2, Figure 3, Figure 4, Figure 5, Figure 6, Figure 7, Figure 8, Figure 9 and Figure 10). In addition, for plants and herbal formulations, the families, utilized parts, tested fraction, and concentrations were mentioned (Table 3). The current work presents different natural compounds with fascinating skeletons that could be effective for Con A-induced hepatitis. This could attract the interest of pharmacologists and medicinal chemists and offer valuable insights for treating AIH. Therefore, these metabolites could be promising prototype compounds for the discovery of drug candidates that could be used for AIH treatment. Also, this could provide new strategies for immune-mediated liver injury treatment.

## 2. Natural Compounds with Hepato-Protective Effect on Con A-Induced Injury

### 2.1. Alkaloids

Sophocarpine, a matrine-type alkaloid reported from *Sophora tonkinensis*, reduced total bilirubin (TBIL) and aminotransferase serum levels and alleviated liver fibrosis by inhibiting the toll-like receptor-4 (TLR4) pathway [31]. Its protective mechanisms against Con A-induced liver injury were assessed by measuring IFN-γ, TNF-α, IL-10, and transforming growth factor-β1 (TGF-β1) serum levels by ELISA and the % of CD4^+^IL-4^+^ and CD4^+^IFN-γ^+^ in the splenocytes of mice by flow cytometry. Sophocarpine at doses 30 and 60 mg/kg inhibited the production of IFN-γ and TNF-α and decreased the % of CD4^+^IFN-γ^+^, while there was no significant effect on the % of CD4^+^IL-4^+^ and TGF-β1 and IL-10 expression. Thus, it attenuated the IFN-γ and TNF-α induced hepatic destruction. Moreover, it markedly lowered expression levels of mRNA of adhesion molecules and chemokines, such as CXC chemokine ligand 10 (CXCL10), intercellular adhesion molecule-1 (ICAM-1), as well as macrophage inflammatory protein-1α (MIP-1α). Expression of T-bet was significantly lowered by an inhibition of activators and signal transducers of transcription-1 (STAT1) activation and increased expression of suppressor of cytokine signaling-1 (SOCS1), inhibiting T-helper type 1 (Th1) cells activation, and IFN-γ expression. Thus, its protective effect was linked to suppressed chemokines, pro-inflammatory cytokines, and signaling of IFN-γ/STAT1 [10].

The isoquinoline alkaloid, demethyleneberberine (DMB) (doses 7.5, 15, and 30 mg/kg) isolated from *Phellodendri chinensis* protected liver architecture from injury induced by Con A- by lowering hepatohemia, necrosis of liver cells, inflammation, as well as lactate dehydrogenase (LDH), alanine transaminase (ALT), and aspartate transaminase (AST) in the blood. Another effect is the lowered liver oxidative stress by reducing malondialdehyde (MDA) level and increasing GSH level in the liver; therefore, it possessed a significant hepato-protective effect in AIH. DMB significantly reduced inflammatory cytokines’ expression and release, namely: TNF-α, IL-6, IFN-γ, and IL-1β as well as the CD4^+^ T cell infiltration and KCs. DMB inhibited nuclear factor κB (NF-κB) signaling activation via prohibiting IκBα, NF-κB p65, and IKKα/β phosphorylation and degradation of IκBα. Furthermore, mitogen-activated protein kinases (MAPKs) and signal transducer and activator of transcription 3 (STAT3) were inhibited. Regulation of MAPK and NF-κB signaling are thought to underlie DMB protection against Con A-induced AIH [9].

The isoquinoline alkaloid, berberine, obtained from *Phellodendron amurense* and *Coptis chinensis* significantly reduced serum AST and ALT levels at conc. 100 or 200 mg/kg in mice with Con A-induced hepatitis. Moreover, it reduced leukocyte infiltration, hepatocyte swelling, and cell death. IL-2, TNF-α, IFN-γ, and IL-1β were lowered, but there was an elevation in IL-10 caused by berberine [21]. Furthermore, berberine significantly increased the ratio of phosphorylated acetyl coenzyme-A carboxylase (ACC) to total ACC, indicating that berberine activated the signaling of adenosine 5‘-monophosphate-activated protein kinase (AMPK) [21].

Zhang et al. [4] found that capsaicin (1 mg/kg) markedly decreased the elevation in ALT level and prevented the apoptosis induced by Con A. Also, it markedly downregulated Bcl-2 associated X protein (Bax) mRNA and upregulated B-cell lymphoma 2 (Bcl-2) mRNA. It improved superoxide dismutase (SOD) activity, decreased MDA and IFN-γ levels, alleviated myeloperoxidase (MPO) high level, and suppressed TNF-α secretion. Furthermore, it suppressed T cells (CD3^+^) activation. Interestingly, the observed higher CD11b^+^Gr-1^+^ MDSCs percentage in the spleen and liver suggested that capsaicin recruited more myeloid-derived suppressor cells (MDSCs) to reduce liver injury [4].

Tetrandrine (TET, bis-benzylisoquinoline alkaloid) reported from *Stephania tetrandra* markedly reduced ALT release by 90% at dose 25 mg/kg and almost totally suppressed at 50 mg/kg. It inhibited the activation and infiltration of the liver via decreasing intrahepatic leukocytes (IHLs) and suppressing the expression of CD69 (T-activation marker). The Con A-induced rise in IFN-γ, TNF-α, IL-4, and IL-12 plasma concentrations were dramatically attenuated as well as MIP-1α and IFN-inducible protein-10 (IFN-γ-IP-10) expressions were decreased by TET. Furthermore, TET inhibited NF-κB activity by prevention of IκBα kinase-α (IKKα) activation and then inhibition of IκBα phosphorylation to alleviate IκBα in liver leukocytes [32].

Fumigaclavine C, an alkaloid was biosynthesized by *Aspergillus fumigatus*. It induced a dose-dependent inhibition of the elevation in transaminases (ALT and AST) activities and increased mice survival rate. It prohibited TNF-α and IL-2 synthesis and spleen cell proliferation. Moreover, it inhibited spleen cells adherence to fibronectin, laminin, and type IV collagen [33].

Halofuginone (HF) (10 ppm) from *Dichroa Febrifuga* significantly decreased the elevated levels of TGF-β1, procollagen III (PCIII), transaminases (AST and ALT), and hyaluronic acid (HA) induced by Con A. It also lowered levels of Smad3, alpha-smooth muscle actin (α-SMA), collagen I, tissue inhibitors of metalloproteinase 2 (TIMP2) in hepatic tissues. This effect has been linked to an attenuation of the TGF-β1/Smad3 signaling pathway. The extent of liver fibrosis was markedly reduced by H. It also decreased the synthesis of the inflammatory markers (IL-1β, TNF-α, and IL-6) and nuclear factor κB (NF-κB). This indicated that HF attenuated the liver fibrosis induced by Con A by lowering the synthesis of collagen I and reducing the liver injury due to inflammation [34].

Secoemestrin C potently inhibited Con A-induced splenocytes proliferation and upregulated expression of these suppressors of cytokine signaling (SOCS) proteins in splenocytes. It also inhibited proliferation and proinflammatory cytokines (IFN-γ, ILs-2, -4, -6, and -17, and TNF-α) secretion of hepatic mononuclear cells (MNCs) in vitro. Secoemestrin C (10 mg/kg) almost completely inhibited the increase in ALT, AST, and LDH due to Con A. Stimulation of both NKT and conventional T cells due to Con A was markedly reduced by secoemestrin C as well as a lowered production of IFN-γ production by both cell types. Moreover, it markedly prohibited the increase in CD69 expression on hepatic NKT cells and conventional CD4^+^ and CD8^+^ T cells [35].

An oxepine-containing diketopiperazine alkaloid, varioxepine B (conc. 10 mg/kg) biosynthesized by *Aspergillus terreus* was evaluated for its immunosuppressive activity in Con A (conc. 15 mg/kg) induced AIH. Liver injury markers: AST, ALT, LDH, direct bilirubin (Dbil), and total bilirubin (TBil) were assessed in the serum and found to be significantly reduced by varioxepine B. Active caspase-3 staining and TUNEL staining revealed that varioxepine B decreased hepatocellular apoptosis, also it markedly lowered IL-2, TNF-α, and IFN-γ in the serum by using a cytometric bead array (CBA) kit. More importantly, when studied in anti-CD3/anti-CD28 monoclonal antibodies (mAbs)-induced murine splenocytes it was found to have an inhibitory effect. Furthermore, it caused an inhibition of T-cell proliferation in humans and resulted in an inhibition of anti-CD3/anti-CD28 mAb-induced human Th1 and Th2 cytokine synthesis [20].

Zhao et al. [36] reported that nicotine (conc. 0.5 and 1 mg/kg) lowered AST and ALT levels significantly, ameliorated pathological lesions, and lowered TNF-α, IL-1β, IFN-γ, and IL-6 and chemokines ((C-C motif chemokine ligand 5 (CCL5), CXCL10, C-X-C motif chemokine 2 (CXCL2), and intercellular adhesion molecule 1 (ICAM-1)) expression induced by Con A (15 mg/kg). Interestingly, it also resulted in an inhibition of inflammation due to Con A in primary cultured cells KCs without affecting splenocytes proliferation. KC-depleted mice did not exhibit the same effects, suggesting that KCs are required in this process. Moreover, nicotine dramatically increased α7-nicotinic acetylcholine receptors (α7-nAChR) expression on KCs, which was reversed by methyllycaconitine (MLA, a specific α7-nAChR antagonist). Thus, it increased α7-nAChR-mediated muscarinic effects in KCs, causing a reduction of Con A-induced AIH via decreasing signaling of NF-κB [36].

Wang et al. [37] isolated two new alkaloids soyalkaloid A and isoginsenine along with four known ones: ginsenine, (1S,3S)-1-methyl-1,2,3,4-tetrahydro-β-carboline-3-carboxylic acid, (1R,3S)-1-methyl-1,2,3,4-tetrahydro-β-carboline-3-carboxylic acid, and indole-3-carboxylic acid from fresh fruits of soybean (*Glycine max*)**.** These metabolites (conc. 0, 0.25, 0.5, 1.0, and 2.0 μM/mL) were assessed for their inhibitory activities on Con A-activated lymphocytes proliferation (conc. 10 μg/mL) using cell counting kit-8 (CCK8) assay. Only soyalkaloid A significantly reduced the proliferation of Con A-activated lymphocytes at conc. 0.5 to 2.0 μM/mL. Its inhibitory effect at conc. 2.0 μM/mL was similar to cyclosporine A (immunosuppressant). However, the other alkaloids had no significant immunosuppressive activity [37].

### 2.2. Terpenes

Liu and Mao [8] evaluated the hepato-protective effect of the monoterpene, eugenol (4-allyl-2-methoxy phenol) in Con A-induced hepatitis. The pretreatment with eugenol significantly lowered ALT and AST activities, with a concurrent reduction of thiobarbituric acid reactive substances (TBARS) level in the liver. It also increased liver metabolic function, observed as increased albumin and decreased bilirubin levels in the serum. Moreover, it increased the activity of antioxidant enzymes (glucose-6-phosphate dehydrogenase (G6PDH), GSH-regenerating enzyme, and glutathione reductase (GR). It had anti-inflammatory and anti-fibrogenic effects as it reduced interleukins (Il-6 and IL-1β) and TNF-α levels. Additionally, it decreased mitochondrial oxidative stress and restored mitochondrial redox balance which was elevated in hepatitis. Eugenol exerted a protective effect through modulation of cytokines‘ levels and inhibition of mitochondrial oxidative stress [8].

Chen et al. [38] reported that the pretreatment with the monoterpene glucoside, paeoniflorin (PF) (50 mg/kg) caused a significant lowering of elevated ALT and AST levels in the liver and necrosis due to Con A. Also, a suppressed content of IL-6, INF-γ, TNF-α, and decreased NKT, CD8^+^, and liver infiltration of CD4^+^ cells was noted with PF. In addition, expression of TLR-4 protein and mRNA was reduced in the liver. Moreover, it suppressed NF-κB activation by IκBα kinase inhibition and phosphorylation of p65 in liver injury related to Con A. Its protective effect against liver damage due to Con A was related to the inhibition of NF-κB activation and downregulation of TLR4 expression [38].

The sesquiterpene lactone, parthenolide, remarkably reduced the congestion and necro-inflammation of the livers induced by Con A. In addition, it suppressed the F4/80^+^ macrophage infiltration, CD49b^+^ NK cells, and hepatic CD4^+^ T cells. Meanwhile, it restored liver function as evidence by lowered serum ALT and AST activities and promoted expression of Ki67. Moreover, it decreased STAT3 and p38 phosphorylation and promoted the p53 phosphorylation in RAW264.7 cells in vitro. Thus, its possible molecular mechanism is suppressing the STAT3/p38 signaling and upregulating the p53 signals [39].

Betulin, a pentacyclic triterpenoid was isolated from *Hedyotis hedyotidea* by [40]. Betulin (16 and 32 μg/mL) induced in vitro dose-dependent suppression of Con A (5 μg/mL)-stimulated mouse splenocytes proliferation using CSFE-dilution assay. At a dose of 20 mg/kg, it significantly inhibited the elevation of ALT and AST in the blood and reversed the effects of Con A in the liver of mice. Also, it decreased IL-6, IFN-γ, and TNF-α induced by the Con A challenge. Furthermore, a significant inhibition of the Con A-related NKT and conventional T-cells activation and downregulated TNF-α, IL-6, and IFN-γ production in these two cells [40].

Zhou et al. [41] stated that (5R)-5-hydroxytriptolide (LLDT-8) markedly attenuated serum ALT level and hepatic necrosis and increased survival rate. LLDT-8 reduced TNF-α, IFN-γ, IL-2, -12, and -6. It also eliminated T cell activation due to an increase in the STAT1 and IRF-1 proapoptotic genes spleen expression. The expression of IFN-γ-IP-10, monokine induced by IFN-γ (Mig), IFN-inducible T cell-α chemoattractant (I-TAC), and vascular cell adhesion molecule-1(VCAM-1) and chemokine receptors (C-X-C chemokine receptor 3 (CXCR3), C-C chemokine receptor 1 (CCR1), and C-C chemokine receptor 5 (CCR5)) mRNA was also inhibited in the hepatic tissues. Therefore, IFN-γ/STAT1/IRF-1 release of cytokines as well as signaling was attenuated significantly by LLDT-8 [41].

Pristimerin (Pris) a quinonemethide triterpenoid, was given at 0.4 and 0.8 mg/kg before Con A (15 mg/kg) administration. After Pris was administered, hepatic damage due to Con A was improved and an attenuation of the raised levels of ALT, ALP, AST, and LDH was noted. In addition, hepatic histopathology was ameliorated after Pris administration. Expression levels of MPO were also improved after Pris, resulting in a reduction in neutrophil infiltration due to Con A. Furthermore, a decrease of lipid peroxidation and an attenuation of Con-A related raised CD4^+^ T cells in the liver was observed. *m*RNA expression of nuclear factor erythroid 2-related factor2 (Nrf2) was enhanced by Pris and it also resulted in higher binding capacities. Heme-oxygenase-1 (HO-1) mRNA were also found to be higher after Pris administration and were restored to their control levels. NF-κB levels and expression were also lowered, in addition to the mediators of inflammation IL-1β, TNF-α, and IL-6. Moreover, it inhibited Con A-induced alteration in apoptosis in hepatic tissues. This was due to a reduction of the expression of mRNA of capsase-3 and Bax as markers of apoptosis and a raise in the expression of mRNA of Bcl2 as an anti-apoptosis marker [12].

The triterpenoid, glycyrrhizin (GL, 100 mg/mL) ameliorated Con A (150 μg/mL) induced liver injury, which manifested by a lower production of IL-6, IFN-γ, and IL-17 and serum ALT. Interestingly, the endogenous alarming inflammatory molecule, high-mobility group box 1 (HMGB1) was markedly lowered by GL combined with Con A in terms of protein levels and mRNA. In contrast, GL upregulated IL-25 (IL-17E) production, which led to a raised proportion of inflammatory protective lymphocyte, Gr-1^+^ CD11b^+^ MDSCs (myeloid-derived suppressor cell) in the liver to restrain hepatic inflammation due to Con A. Therefore, the protection caused by GL could be linked to a reduction in IL-17 synthesis and an enhanced expression of IL-25 expression [5]. Another study by Tu et al. [42] elucidated the immuno-modulatory actions of GL on CD4^+^T cells of hepatic fibrogenesis. The study revealed that there was a dramatic prevention of hepatic fibrosis and inflammation after GL administration. In addition, infiltration of Th1, Th2, Th17, and regulatory T cells (Treg) was inhibited in the spleen and liver, and a regulation of Th1/Th2 and Treg/Th17 balances was noted, respectively to a relative increase of liver lineages of Th1 and Treg. Also, a major increase of IFN-γ and IL-10 as antifibrotic cytokines was noted after GL administration. Moreover, GL inhibited splenic CD4^+^T cell proliferation due to Con A and increased the mRNAs of IL-10 and IFN-γ in these cells. Moreover, Con A-related phosphorylation of PI3K/AKT, JNK, and ERK was significantly inhibited by GL. A regulatory mechanism of the CD4^+^T cell response in pathways dependent on PI3K/AKT, JNK, and ERK is believed to underlie the alleviated liver damage and progression of fibrosis caused by GL [42].

Glycyrrhizic acid ammonium salt (GAAS), a derivative of glycyrrhizic acid, is commonly used for treating immune-mediated hepatic damage. A study by Tian et al. [43] was carried out to investigate the GAAS mechanism for alleviating immune-mediated hepatic damage due to Con A. It was revealed that GAAS (20 mg/kg) downregulated the expression of IL-1β, -6, and -17A, TNF-α, and IFN-γ mRNA and caused an upregulated expression of TGF-β and IL-4 mRNA. Furthermore, mice studies revealed that the balance of immune cells; Th1, -2, and -17, and Treg was mediated by GAAS after regulation of T-bet, GATA3, RORγt, and Foxp3 expressions to reverse hepatic injury. Moreover, GAAS caused a blockade of the JAK1/STAT1/IRF1 pathway leading to a reduction in apoptosis of hepatocytes, suppressed oxidative stress, and a controlled expression of proteins related to apoptosis [43].

Magnesium isoglycyrrhizinate (MIG), a 18-α glycyrrhizic acid magnesium salt at doses 12.5, 25, and 50 mg/kg before Con A (20 mg/kg) administration significantly reduced serum ALT and AST and alleviated the liver injury and inflammatory cell infiltration. MIG also decreased the levels of MDA, neopterin (NP), and MPO in liver tissues, increased that of SOD, and reduced TNF-α and IFN-γ in the blood [44].

Klein et al. [45] reported that ME3738 (40 mg/kg) pretreatment before Con A (30 mg/kg) caused a marked inhibition of hepatic damage and alleviated increased levels of ALT and AST. It also induced IL-6 and activated STAT3 binding of DNA and transcription of target genes. Therefore, ME3738 triggered the expression of IL-6, which in turn leads to an activation of several pathways offering protection from Con A-related damage [45].

The *Glycine max* triterpenoid, soyasapogenol A (2 mg/mouse) administration before the injection of Con A decreased hepatic inflammatory cells infiltration and significantly lowered raised plasma levels of ALT and TNF-α. Hepatic parenchymal cells also have a lowered number of apoptotic bodies. It was suggested that soyasapogenol A directly prevented the hepatocytes apoptosis and inhibited the rise of TNF-α in the blood, leading to prevention of Con A-related hepatic injury [46].

Cycloastragenol (CAG) significantly downregulated CD25 and CD69 expression on the surface of Con A stimulated CD3^+^ T cells and inhibited proliferation of activated lymphocytes. A blockage of mitogenesis related to Con A was also noted with CAG, whereby an arrest of the lymphocyte G0/G1-phase cell-cycle was noted and a marked reduction of cells in the S and G2/M phase. Furthermore, CAG significantly declined [Ca2^+^]_i_. In addition, the synthesis of Th1 cytokines (IFN-γ, TNF, and IL-2), Th2 cytokines (IL-4, -6, and -10), and Th17 cytokine (IL-17 A) on lymphocytes activated by Con A was inhibited by CAG. Therefore, CAG inhibited lymphocyte proliferation, activation, and cytokines expression, which could be explained by reduced overall intracellular Ca^2+^ levels [47].

Studies with taraxasterol revealed that it resulted in a marked reduction of the Con A-related raised liver indices, ALT, AST, and liver MDA. It also caused enforced the Con A-related reduction of GSH and SOD synthesis. Moreover, an inhibition in the release of the inflammatory cytokines was noted, namely: IL-6, TNF-α, IL-1β, IFN-γ, and IL-4. Furthermore, an alleviation of the liver histo-pathological changes and apoptosis due to Con A was observed. Taraxasterol, resulted in a major downregulation in TLR2, TLR4, and NF-κB p65 expression and decreased the Bax/Bc1-2 expression ratio in hepatic tissues. Accordingly, taraxasterol prevented Con A-related liver damage by inhibition of the TLRs/NF-κB signaling pathway and promoting Bax/Bc1-2 anti-apoptotic signaling pathway [48].

The protection offered by the pregnane glycoside, periplocoside A (PSA, conc. 10 mg/kg), isolated from *Periploca sepium* on Con A-induced hepatic injury was elucidated by Wan et al. [22]. PSA markedly ameliorated Con A (15 mg/kg)-related hepatic damage, which was manifested by decreasing blood ALT, IL-4, and IFN-γ and hindering hepatic apoptosis and necrosis, leading to elevation of the survival rate. It inhibited IFN-γ and IL-4 production of α-galactosylceramide (α-GalCer) or anti-CD3-activated NKT cells in vitro. Moreover, it suppressed IFN-γ translation and IL-4 transcription. The PSA preventative effect was linked to an inhibited production of NKT-derived cytokines [22].

Xie et al. [49] carried out a study to evaluate the potential of platycodin D (PD) as an alternative addition to Alum in vaccinations of hepatitis B. They used a formulation comprised of hepatitis B surface antigen (HBsAg) with Alum and PD on mice. PD treated mice displayed an increased splenocyte proliferation due to Con A-, HBsAg, and LPS. HBsAg-immunized mice showed increased antiserum titers of HBsAg-specific IgG, IgG1, IgG2a, and IgG2b antibodies. In addition, Th2 (IL-10) and Th1 (IL-2 and IFN-γ) cytokines production was greatly increased due to PD and the expression of Th1 cytokines in HBsAg-immunized mice was found to be upregulated in splenocytes. An enhancement in the killing activity of cytotoxic T lymphocytes (CTLs) and NK cells was noted with PD. Therefore, PD increased both humoral and cellular immune reactions and elicited a controlled Th1/Th2 response counter to HBsAg [49].

Also, in 2010 Xie et al. evaluated the potential of platycodin D2 (PD2) in enhancing specific humoral and cellular immune reactions to HBsAg. When studies on mice immunized with HBsAg, PD2 resulted in a significant rise in the Con A-, LPS-, and HBsAg-related proliferation of splenocytes. It markedly increased HBsAg-specific IgG1, IgG, IgG2b, and IgG2a antibody concentrations in the serum. In addition, PD2 significantly increased synthesis of Th2 (IL-10 and IL-4) and Th1 (IFN-γ and IL-2) cytokines in the HBsAg-immunized mice splenocytes. The PD2 adjuvant potential on proliferation of splenocytes, blood HBsAg-specific IgG2a and IgG2b responses, and secretion of Th1-cytokine was higher than that of Alum. PD2 improved both humoral and cellular immunity with dual Th2/Th1-potentiating effect in the HBsAg-immunized mice [50].

Stephanthraniline A (STA) resulted in a significant reduction in the Con A-related liver damage and a reduction in the aggregation and activation of CD4^+^ T cells in the liver. It caused a direct reduction in the proliferation and activation of Con A-related CD4^+^ T cells and an inhibition of microtubule-associated protein kinase (MAPK), NFκB, and nuclear factor of activated T-cells (NFAT) pathways in CD4^+^ T cells. Also, T cells activation was arrested by STA, as well as the proliferation via proximal T-cell receptor (TCR) signaling- and Ca^2+^ signaling. STA also resulted in a direct inhibition of protein kinase Cθ (PKCθ) activity as well as its phosphorylation CD4^+^ T cells, which are activated. Therefore, STA protected from CD4^+^ T cell-related immune hepatitis via PKCθ and the signaling pathways of NFAT, NFκB, and MAPK [51].

### 2.3. Sterols

Fucosterol is a sterol found in the brown alga *Eisenia bicyclis*. Fucosterol pretreatment (Conc. 25, 50, and 100 mg/kg) attenuated serum liver enzymes (ALT and AST) and liver cell apoptosis and necrosis and apoptosis due to TNF-α, IL-6, and IL-1β in BALB/c mice which were given Con A (25 mg/kg). Fucosterol also resulted in an upregulation of Bcl-2, resulting in lower levels of Beclin-1 and Bax ultimately leading to an inhibition of autophagy and apoptosis. It also caused an activation of PPARγ and a reduction in the NF-κB p65 and P38 MAPK signaling. Hence, fucosterol inhibited P38 MAPK/NF-κB/PPARγ signaling and caused an alleviation of Con A-related hepatic injury [13].

Chen et al. [53] studied the protection offered by zhankuic acid A (ZAA) in Con-A- induced hepatitis in mice. They reported an inhibition of stimulator-activated T cell proliferation by ZAA. Blood concentrations of IFN-γ, IL-6, and IL-4 were diminished by 37%, 66% and 38%, respectively. Furthermore, ZAA lowered the expression of interferon regulatory factor-1 (IRF-1) expression and the liver and spleen activity of caspase-3. Moreover, ZAA (20 mg/kg) resulted in lowered leukocyte infiltration and necrosis. It also restored AST and ALT levels. A reduction in the downstream signaling of Janus kinase 2 (JAK2) and its phosphorylation as well as an inhibition of the IFN-γ/STAT-1/IRF-1 pathway was also noted with ZAA [53].

When ursodeoxycholic acid (UDCA) was administered prior to Con A (20 mg/kg) at doses of 50 and 150 mg/kg, a reduction in the raised AST and ALT levels was found as well as a lower occurrence of hepatic necrosis. No change in the concentration of hepatic hydrophobic bile acids was found. Elevated levels of macrophage inflammatory protein-2 (MIP-2), IL-6, and TNF-α in the blood were markedly inhibited by UDCA. It also resulted in a significant reduction in the liver concentrations of TNF-α and MIP-2. A reduction in MPO activity and MIP-2 levels in the liver was also noted with an accumulation of neutrophils intra-sinusoidally. UDCA also halted the synthesis of MIP-2 and TNF-α in cultures with lymph node and non-parenchymal cells [52].

### 2.4. Phenolic Compounds

Hu et al. [54] stated that pretreatment with salidroside (50 mg/kg) dramatically lowered ALT and AST and the extent of liver necrosis. Also, it suppressed inflammatory cytokines (IFN-γ, TNF-α, and IL-6) secretion via lowering NF-𝜅B activity. In addition, it altered the CD8^+^ and CD4^+^T lymphocyte hepatic and spleen distribution via CXCL-10 regulation, which lowered liver injury severity [54].

Huang et al. [80] investigated the effect of resveratrol (30 mg/kg) on Con A (20 mg/kg)-induced hepatitis in aged mice. Resveratrol dramatically decreased elevated levels of TNF-α, IL-6, IFN-γ, and monocyte chemoattractant protein-1 (MCP-1), and infiltration of T-lymphocytes, neutrophils, and macrophages in the liver induced by Con A. It significantly reduced raised MDA levels and reversed the decreased activities of GSH and SOD and the levels of glutathione S-transferase (GST) and manganese superoxide dismutase (MnSOD) in livers of aged mice challenged with Con A. It reversed the Con A-inhibition of proliferating cell nuclear antigen (PCNA), Ki67, p-cyclin-dependent kinase 2 (p-Cdk2), and cyclin D1 expressions in the livers. Moreover, it reduced the raised hepatic p66^shc^ by causing a reversal of downregulated SIRT1 due to Con A. Thus, resveratrol protected aged mice from hepatitis due to Con A by reducing the irregular liver regeneration and immune responses, by partly acting via SIRT1-mediated repression of p66^shc^ expression mechanism [80]. Moreover, Zhou et al. [55] reported that the pretreatment with resveratrol (30 mg/kg) ameliorated the pathologic effects of autoimmune hepatitis in Balb/C mice due to Con A (20 mg/kg). It caused a marked reversal of the raised ALT and AST, inhibited TNF-α, IL-2, and IL-6 production, and attenuated glioblastoma-1 (Gli-1), sonic hedgehog (Shh), and patched (Ptc) expression. Resveratrol protected from Con A-related AIH by lowering the expression of cytokines and Gli-1 and Ptc release via acting on the hedgehog signal cascade [55].

Several studies have been carried out to elucidate the protective effects and the possible mechanisms of curcumin (diferuloymethane), a yellow-colored polyphenol from *Curcuma longa* on Con A-related liver damage. Wang et al. [56] reported that curcumin (100 mg/kg) pre-treatment, caused a significant attenuation of the raised ALT level. It further reduced liver necrosis, apoptosis, and mortality. A marked reduction of oxidative stress in the liver was noted (↓ MDA and ↑ GSH levels) as well as TNF-α and IFN-γ. HMGB1release in hepatic tissues was also significantly lower after curcumin administration. The protective effect of curcumin was a result of lowered hepatic oxidative stress, inflammation, and release of HMGB1 [56]. Tu et al. [11] reported that curcumin (conc. 200 mg/kg) resulted in a marked inhibition of plasma ALT and AST, suppressed production of TNF-α, IL-4, and IFN-γ, and lowered recruitment of CD4^+^ T lymphocytes to hepatic tissues due to Con A (20 mg/kg). When studied in a BALB/c mouse model of Con-A induced liver damage, curcumin was found to inhibit the expression of intrahepatic intercellular adhesion molecule-1 (ICAM-1) and CXCL10 [11]. Another work by Tu et al. [81] found that pre-treatment with curcumin resulted in lower liver expression of TNF-α and IFN-γ genes and higher IL-10 expression. Furthermore, it significantly lowered hepatic mRNA and protein concentrations of TLR2, 4, and 9. This inhibition in the expression of TLR2, 4, and 9 could underlie the cucumin-induced hepato-protective effect [81]. In 2013, Tu et al. reported that curcumin significantly decreased serum levels of HMGB1. Also, a reduction of nucleus to cytoplasm translocation of HMGB1 was reported in hepatocytes. Furthermore, hepatic mRNA and protein expression of HMGB1 were also reduced. In addition, it inhibited the intrahepatic protein expression of IL-6, TNF-α, and IL-1β. This could be due to a partial reduction of translocation of hepatocyte HMGB1, blood release, and liver expression [82].

The citrus flavonoid, hesperidin (HDN) showed protective effects on Con A (15 mg/kg)-induced hepatitis in male C57BL/6 mice. Pre-treatment with HDN (1000 mg/kg) resulted in a reduction of Con A-related ALT and AST elevations. Furthermore, it decreased oxidative stress in the liver (↓ MDA and ↑ GSH levels) and the synthesis of cytokines (TNF-α and IFN-γ). It markedly reduced the expression and releasing of HMGB1 and T-cell activation. Thus, HDN suppressed cytokines production, hepatocyte oxidative stress, releasing and expressing HMGB1, and T cells activation [57].

Pre-treatment with quercetin (50 mg/kg) resulted in a reduction in Con A (20 mg/kg)-related raised ALT and AST and necrosis in the liver. It also caused a reduction in blood levels of the pro-inflammatory cytokines (TNF-α, INF-γ, and IL-4). A lowered HMGB1, TLR2, and TLR4 mRNA and protein expression was also observed in hepatic tissues. It also caused a marked degradation of IκBα and modulated Con A-induced nuclear translocation of NF-κB p65in the liver. The protective potential of quercetin could be linked to a reduction in HMGB1-TLRs-NF-κB signaling [58].

Liu et al. [59] found that baicalin (BA) (200 and 100 mg/kg) caused a marked lowering of ALT and AST activities, ameliorated apoptosis of hepatocytes, and reversed the raised plasma cytokines (TNF-α, IL-6, and INF-γ) due to Con A (20 mg/kg). Furthermore, BA decreased tissue MPO and caspase 3 activities and lipid peroxidation (↓ MDA level), however it increased the SOD level. The beneficial effect of BA was associated with inhibited TNF-α-induced hepatic apoptosis and reduced production of cytokines from lymphocytes [59].

Baicalein (BE, 5,6,7-trihydroxyflavone) (100 mg/kg) which is obtained from *Scutellaria baicalensis* root was found to alleviate Con A (15 mg/kg)-related liver damage. It reduced TNF-α and IFN-γ blood levels and caused less mononuclear cells (MNCs) infiltration in C57BL/6 mice. Treatment with BE resulted in greater apoptosis in MNCs infiltrating the liver and in splenocytes, as and in CD3^+^ and CD19^+^ splenocytes. In addition, in vitro culture of liver MNCs and splenocytes showed enhanced apoptosis after BE-treatment. Therefore, BE caused an enhancement in apoptosis of activated lymphocytes by acting on mitochondrial signaling [60].

A study by Liu et al. [61] revealed that (-)-epigallocatechin-3-gallate (EGCG) (5 mg/kg), a polyphenolic compound from green tea effectively increased survival rate, ameliorated liver pathology, and lowered ALT in mice treated with Con A (15 mg/kg). TNF-α, IL-6, IL-4, and INF-γ release in the blood was significantly inhibited by EGCG. Levels of MDA were reduced, and GSH and SOD in hepatic tissues due to Con A were corrected. Finally, NF-κB activation and TLR2, TLR4, and TLR9 hepatic protein expression was much lower after EGCG pre-treatment. EGCG possessed protective properties against Con A-related hepatic damage by decreasing oxidative stress, inflammatory cytokine, and less activation of NF-κB as well as expression of TLR [61]. Moreover, it has been noted that EGCG (5 mg/kg) pretreatment prior to Con A (15 mg/kg) injection reduced hepatocyte apoptosis, inflammatory infiltration, and ALT levels (80% reduction) in plasma in the liver. EGCG abrogated TNF-α and INF-γ at the mRNA and protein levels in hepatic tissues. Nitrite blood levels and inducible nitric oxide synthase (iNOS) synthesis in the liver were simultaneously lower. EGCG also resulted in a marked reduction of IP-10 and MIP-1α hepatic expression. Therefore, EGCG has the propensity to control immune-mediated hepatic damage by suppressing the production of several inflammatory mediators [61].

Luo et al. [62] reported that galangin (3,5,7-trihydroxyflavone) from *Alpinia officinarum* at conc. 25 and 50 mg/kg dramatically attenuated raised blood levels of IFN-γ, TNF-α, and Il-12 and decreased chemokines and adhesion molecules including MIP-1α, IP-10, and ICAM-1 messenger RNA expressions in the liver of C57BL/6 mice with concanavalin induced hepatitis (CIH). Moreover, it also decreased T-cell activation and leukocyte infiltration in the liver. Furthermore, it effectively reduced phosphorylation of STAT1 and IκBα, indicating a reduction of IFN-γ and STAT1 signaling due to galangin. Thus, galangin could be modulating essential signaling pathways of inflammation, such as IFN-γ/STAT1 and NF-κB [62].

Studies have shown that pre-treatment with scutellarin 100 mg/kg caused a marked reduction of hepatic lesions due to Con A (25 mg/kg) and also decreased the serum AST, ALT, TNF-α, and NO_2_^−^/NO_3_^−^ levels. Furthermore, it resulted in a downregulation of iNOS and TNF-α mRNA expression as well as c-Fos, c-Jun, and iNOS proteins, in addition to inhibiting IκBα degradation in Con A-injected mice livers. The suggested mechanism underlying the effects of scutellarin was through reducing NF-κB/TNF-α/iNOS signaling [63].

Wang et al. reported that astilbin, a flavanone, isolated *Smilax glabra*, causing a marked inhibition of the elevated activities of ALT and AST as well as reducing TNF-α synthesis. At concentrations of 40 mg/kg, it ameliorated liver histology, including hepatocyte necrosis and inflammatory infiltration, degeneration, and KCs hyperplasia. Furthermore, astilbin halted spleen cells and purified T lymphocytes adhesion to fibronectin, laminin, and type IV collagen in injured mice. Human Jurkat T cells adhesion to endothelial cell line ECV-304 was further halted by astilbin. Accordingly, astilbin reduced the synthesis of TNF-α and the adhesion properties of T cells [64].

Dihydroquercetin (DHQ, 5 mg/kg) significantly ameliorated Con A (30 mg/kg) related liver injury in C57BL/6 (B6) mice by lowering AST and ALT level, protecting against histological alterations, increasing the survival rate, and decreasing IL-2, -4, and -10, TNF-α, IFN-γ, OPN, and iNOS mRNA expression in hepatic tissue. In addition, DHQ significantly enhanced in a time- and dose-related fashion expression of heme oxygenase-1 (HO-1) via increasing cytoplasmic expression of Nrf2 as well as nuclear translocation. Moreover, increased phosphorylation of three members of the MAPK family (p38, ERK, and JNK) was noted with DHQ. The hepato-protective potential of DHQ in Con A-related hepatic damage was attributed to its ability to inhibit the release of inflammatory mediators and to scavenge oxidative stress via upregulating HO-1 action in macrophages/Kupffer cells via MAPK/Nrf2 signaling [65].

It has been stated that shikonin (7.5 and 12.5 mg/kg) caused a marked improvement of hepatic damage, reduced liver necrosis, and lowered inflammatory cytokines’ release (IFN-γ, TNF-α, and IL-1β) in Balb/C mice induced by Con A (20 mg/kg). Bcl-2, caspase 9, and Bax expressions were significantly altered by shikonin, where it downregulated Bax and caspase 9 and caused an upregulation of Bcl-2. Moreover, it decreased p-JNK, microtubule-associated protein 1 light chain 3 (LC3), and Beclin-1 expression levels. Shikonin attenuated Con A-related hepatic damage by lowering autophagy and apoptosis via JNK pathway inhibition [66].

Research reported by Zhang et al. [67] stated that magnolol (doses 10, 20, 30 mg/kg) a biphenyl derivative from *Magnolia officinalis* displayed potential effect on immune-related liver fibrosis BALB/c mice administered Con A (8 mg/kg). It restored liver function, inhibited the raised ALT and AST activity while attenuating liver fibrotic damage in vivo. It inhibited Th17 cell differentiation in Con A-treated liver and suppressed IL-17A production. Also, it suppressed the abnormal hepatic stellate cells (HSCs) activation at doses 20 and 30 mg/kg. Furthermore, it inhibited Smad3 phosphorylation and blocked the Smad3/Smad4 signaling pathway activation in LX2 cells, by upregulating phospho-Smad3 and prohibiting contact between Smad3 and Smad4 leading to partly inactivation of LX2 cells. Its protective actions could be due to its inhibition in HSCs of Th17 cell-mediated Smad 3/4 signal transduction [67].

Pterostilbene (PTE) is a stilbene derivative commonly found in berries. PTE (doses 10 and 40 mg/kg) prior to Con A (10 mg/kg) administration showed a marked and dose-related reduction of blood liver enzymes and alleviated the hepatic necrosis and apoptosis via upregulation of the nuclear antigen Ki67. PTE suppressed the cytokines IFN-γ and TNF-α production in addition to reducing intrahepatic hyper-coagulation. Furthermore, it reversed MAPK phosphorylation (p38, ERK1/2, and JNK) and NF-κB p65 induced by Con A. Interestingly, PTE stopped the Con A-related hepatic injury by acting on the macrophages rather than hepatocytes, where it resulted in an inhibition of accumulation of macrophages in the liver and generation of tissue factor (TF) via halting MAPK p38 activation [68].

A study by Xu et al. [69] reported that the pretreatment with phenolic carboxylic acid derivative, salvianolic acid A (SalA) from *Salvia miltiorrhiza* alleviated Con A-related hepatic damage in Kunming mice. SalA significantly reduced elevations in ALT and AST activities and decreased IFN-γ and TNF-α. Moreover, an improvement in cleaved caspase-3 and NF-κB due to Con A was noted; whereas it reversed β-cell lymphoma-extra-large (Bcl-xL) expression and increased expression of SIRT1. This was linked to p66shc mRNA and protein downregulation. Furthermore, it increased SIRT1 expression in HepG2 cell culture, which was halted by SIRT1 siRNA knockdown. SalA was a potent SIRT activator and alleviated Con A-related hepatic damage via repressing the p66shc pathway by SIRT1 [69].

It was reported that the pretreatment with chlorogenic acid (CGA, doses 3, 10, and 30 mg/kg) prevented the rise in plasma AST and ALT and caused an alleviation of liver histopathological changes and apoptosis of hepatocytes induced by Con A (20 mg/kg). In addition, it markedly suppressed TNF-α and IFN-γ production, reduced neutrophil, activated CD4^+^ T lymphocytes, and hepatic macrophage infiltration. Moreover, it downregulated liver adhesion molecules (ICAM-1, VCAM-1, and ELAM-1) expression of protein and mRNA, and stopped Con A-activated TLR4 molecules (TLR4, p-IRAK1, pIκB, and p-p38.). CGA prevented Con A-related hepatic damage by decreasing pro-inflammatory cytokines production, activation of TLR4 signaling, adhesion molecules expression downregulation, and alleviating hepatic leukocytes activation and infiltration [70].

### 2.5. Coumarins and Coumarin Derivatives

Wedelolactone (7.5 and 12.5 mg/kg) a coumarin from *Eclipta prostrata* caused a marked reduction of blood ALT and AST and liver damage severity induced Con A (15 mg/kg) injection. It dramatically attenuated increased serum concentrations of TNF-α, IFN-γ, and IL-6. Additionally, it lowered expressions of CXCL10 as well as ICAM1 and reduced infiltration of leukocytes and activation of hepatic T-cells. Furthermore, it suppressed the activity of NF-κB by inhibiting the Con A-related IκBα and NF-κB p65 phosphorylation. Thus, its protective effect is mediated by attenuating the modulation of NF-κB signaling [71].

Okamoto et al. [72] investigated the properties of coumarin derivatives, osthole, imperatorin, Pd-Ia, Pd-II, and Pd-III on Con A (0.2 mg/mouse)-related hepatic damage in mice. They reported an inhibition of over 90% of the Con A-related increase of ALT activity after coumarin administration in comparison to glycyrrhizin (45%, 200 mg/kg) at a dose 200 mg/kg; however, osthole had the potent inhibitory effect among the tested coumarins at dose 100 mg/kg [72].

### 2.6. Miscellaneous

The iridoid aglycone, genipin reported from *Gardenia jasminoides* suppressed IL-1β, IFN-γ, IL-12p70, and IL-6 synthesis in Con-A treated splenocytes in culture. Also, it inhibited IFN-γ-stimulated macrophages derived nitrite release [74,83].

Alpha-lipoic acid (α-LA), a key energy metabolism regulator in mitochondria, caused a marked decrease in AST and ALT levels and a reduction of increased blood TNF-α, IL-6, IFN-γ, and IL-10 levels induced by Con A injection, as well as it alleviated histo-morphological changes and hepatic necrosis severity. In addition, α-LA increased GSH and SOD levels but reduced lipid peroxidation and MPO activity in hepatic tissues. It also prevented IκBα, NF-κB p65, and JNK phosphorylation. Thus, α-LA reduced liver damage due to Con A-induced by reducing ROS generation as well as modulating cytokines secretion [75].

Bruck et al. [76] examined the ability of allicin (conc. 1.8–2.0 mg/mL), the active component of garlic in the prevention of immune-related Con A (15 mg/kg)-induced hepatic injury in mice. The histo-pathologic injuries to the livers in these mice and the Con A-related rise of ALT and TNF-α levels were much lower in mice pre-treated with allicin. In addition, the nuclear binding capacity of NF-κB and expression of iNOS protein were attenuated by allicin. It also halted the adhesion of T cells to extracellular matrix components and to endothelial cells via mediating TNF-α. Allicin prohibited the expression of ICAM-1 and VCAM-1 on vascular endothelial cells in humans, an effect which is also mediated by TNF-α. Therefore, allicin’s protective effect was probably due to its immune-modulatory actions on adhesion molecules and T cells coupled with NF-κB activation blockade [76].

Ren et al. [77] investigated the protective effects of protectin D1 (PD1), a docosahexaenoic acid product against Con A (30 mg/kg) induced hepatitis. Pre-treatment with PD1 (conc. 10 and 20 µg/kg) demonstrated to significantly inhibited the elevated ALT and AST levels, liver necrosis, and high mobility group B 1 induced by Con A. Furthermore, PD1 prevented TNF-α, INF-γ, and IL1β, -2, and -6 production. In addition, it markedly suppressed hepatic cellular infiltration in NKT, CD4^+^, and CD8^+^ cells. It also suppressed the pyrin domain containing 3 (NLRP3) and Toll-like receptor (TLR) mRNA and protein expression in hepatic tissues. Moreover, the NF-κB-activated CX3CL1/CX3CR1 signaling cascade activation was blocked by PD1 in Con-A related hepatic injury. This protection has been associated with a reduction of TLR4 expression and downregulation of NF-κB activation [77].

Salecan an extracellular β-glucan that is soluble in water and obtained from *Agrobacterium* sp. (conc. 20 and 40 mg/kg). When studied in C57BL/6 mice, salecan significantly decreased blood activities of AST and ALT and reduced the secretion of inflammatory cytokines (IFN-γ, IL-6, and IL-1β) and their expression in Con A-related hepatic injury model (conc. 20 mg/kg). It downregulated Con A-induced increased chemokine and adhesion molecules expression, including macrophage inflammatory protein-1β (MIP-1β), MIP-1α, MCP-1, ICAM-1, and RANTES. Moreover, an inhibition of T cell activation and infiltration was also noted. A reversal of the metabolic effects in Con A-treated mice towards the control group was also noted and a partial recovery of Con A-related metabolic disturbances was observed [78].

Fucoidan (conc. 1–30 mg/kg) pretreatment prevented Con A (conc. 18.5 mg/kg)-induced rise in ALT, TNF-α, and IFN-γ levels. In addition, it reduced midzonal hepatocellular necrosis and sinusoidal congestion in the liver seen in Con A injected animals. A significant rise in IL-10 synthesis was also observed in serum and hepatic tissues, implicating the Con-A related hepatic damage was prevented by fucoidan through modulation of IL-10 endogenous synthesis as well as an inhibition of cytokines involved in inflammation [79].

## 3. Conclusions

**Figure 11:**
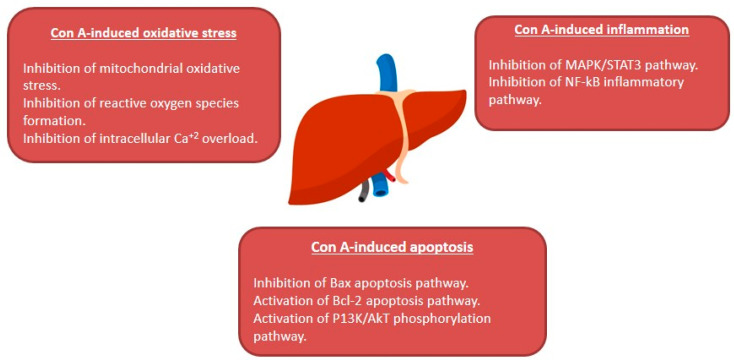
Summary of the hepato-protective mechanisms of natural compounds against Con A-induced hepatotoxicity.

In conclusion, many studies have shown that natural products are effective medicines that could be potentially used to manage liver disorders. AIH represents a major health hurdle that can lead to liver cirrhosis without an available cure. Con A-related hepatic injury is a known and extensively studied model that mimics the histopathological changes of AIH in human. The current review revealed that multi-targets may underlie the protection offered by natural products in Con A-related hepatic injury as summarized in Figure 11. Thus, natural products could provide potential drug targets and promising candidates for the treatment and prevention of AIH. However, the precise mechanism of the protective effects of many reported natural metabolites requires extensive research. Warranting preclinical studies are needed for the metabolites with established protective mechanisms.

## Figures and Tables

**Table 1:** List of natural compounds evaluated for protective effect against Con A-induced liver injury

Compound Name	Class	**Mol. Formula**	**Mol. Weight**	Plant/Fungus Name (Family)	Reference
Alkaloids					
Sophocarpine	Matrine-type alkaloid	C_16_H_24_N_2_O	260	*Sophorae Subprostrata* = *Sophora tonkinensis* (Fabaceae)	[10]
Demethyleneberberine	Isoquinoline alkaloid	C_19_H_18_NO_4_^+^	325	*Phellodendron chinense* (Rutaceae)	[9]
Berberine	Isoquinoline alkaloid	C_20_H_18_NO_4_^+^	337	*Coptis chinensis* (Ranunculaceae) *Phellodendron amurense* (Rutaceae)	[21]
Capsaicin (8-methyl-N-vanillyl-6-nonenamide) *	Phenolic amide alkaloid	C_18_H_27_NO_3_	305	*-*	[4]
Tetrandrine	*bis*-Benzylisoquinoline alkaloid	C_38_H_42_N_2_O_6_	622	*Stephania tetrandra* (Menispermaceae)	[32]
Fumigaclavine C	Ergoline alkaloid	C_23_H_30_N_2_O_2_	366	*Aspergillus fumigatus* (strain No. CY018) (Aspergillaceae)	[33]
Halofuginone	Quinazoline alkaloid	C_16_H_17_BrClN_3_O_3_	413	*Dichroa febrifuga* (Hydrangeaceae)	[34]
Secoemestrin C	Epitetrathiodioxopiperazine alkaloid	C_27_H_22_N_2_O_10_S	566	*Aspergillus nidulans* (B, C) (Aspergillaceae)	[35]
Varioxepine B	Diketopiperazine alkaloid	C_27_H_31_N_3_O_5_	477	*Aspergillus terreus* (strain No. CY018) (Aspergillaceae)	[20]
Nicotine *	Pyridine alkaloid	C_10_H_14_N_2_	162	-	[36]
Soyalkaloid A	Indole alkaloid	C_13_H_10_N_2_O_2_	226	*Glycine max* (Fabaceae)	[37]
Isoginsenine	Azepino-indole alkaloid	C_13_H_14_N_2_O_2_	230	*Glycine max* (Fabaceae)	[37]
Ginsenine	Azepino-indole alkaloid	C_13_H_14_N_2_O_2_	230	*Glycine max* (Fabaceae)	[37]
(*1*S,3*S*)-1-Methyl-1,2,3,4-tetrahydro-β-carboline-3-carboxylic acid	Tetrahydro-β-carboline alkaloid	C_13_H_14_N_2_O_2_	230	*Glycine max* (Fabaceae)	[37]
(1*R*,3*S*)-1-Methyl-1,2,3,4-tetrahydro-β-carboline-3-carboxylic acid	Tetrahydro-β-carboline alkaloid	C_13_H_14_N_2_O_2_	230	*Glycine max* (Fabaceae)	[37]
Indole-3-carboxylic acid	Indole alkaloid	C_9_H_7_NO_2_	161	*Glycine max* (Fabaceae)	[37]
Terpenes					
Eugenol *	Phenolic monoterpene	C_10_H_12_O_2_	164	-	[8]
Paeoniflorin	Monoterpene glucoside	C_23_H_28_O_11_	480	*Paeonia lactiflora* (Paeoniaceae)	[38]
Parthenolide	Sesquiterpene lactone	C_15_H_20_O_3_	248	*Tanacetum parthenium* (Asteraceae)	[39]
Betulin	Triterpenoid	C_30_H_50_O_2_	442	*Hedyotis hedyotidea* (Rubiaceae)	[40]
(5*R*)-5-hydroxytriptolide	Diterpenoid epoxide	C_20_H_24_O_7_	376	*Tripterygium wilfordii* (Celastraceae)	[41]
Pristimerin	Quinonemethide triterpenoid	C_30_H_40_O_4_	464	-	[12]
Glycyrrhizin *	Triterpenoid	C_42_H_62_O_16_	822	*-*	[5,42]
Glycyrrhizic acid ammonium salt *	Triterpenoidal acid salt	C_42_H_62_O_16_.NH_3_	839	-	[43]
Magnesium isoglycyrrhizinate *	Triterpenoidal acid magnesium salt	C_42_H_60_MgO_16_	844	-	[44] [23]
ME3738 *	Triterpenoid	C_31_H_52_O_3_	472	-	[45]
Soyasapogenol A	Triterpenoid	C_30_H_50_O_4_	474	*Glycine max* (Fabaceae)	[46]
Cycloastragenol *	Cycloartane triterpenoid	C_30_H_50_O_5_	*490*	-	[47]
Taraxasterol	Triterpenoid	C_30_H_50_O	426	*Taraxacum officinale* (Asteraceae)	[48]
Periplocoside A	Pregnane glycoside	C_73_H_116_O_27_	1424	*Periploca sepium* (Apocynaceae)	[22]
Platycodin D	Triterpenoid glycoside	C_57_H_92_O_27_	1208	*Platycodon grandiflorum* (Campanulaceae)	[49]
Platycodin D2	Triterpenoid glycoside	C_63_H_102_O_33_	1386	*Platycodon grandiflorum* (Campanulaceae)	[50]
Stephanthraniline A	Polyoxypregnane	C_31_H_43_NO_8_	557	*Stephanotis mucronata* (Asclepiadoideae)	[51]
Sterols					
Fucosterol *	Steroid	C_29_H_48_O	412	*-*	[13]
Ursodeoxycholic * acid	Steroid	C_24_H_40_O_4_	392	-	[52]
Zhankuic acid	Steroid acid	C_29_H_42_O_6_	478	*Taiwanofungus camphoratus*	[53]
Phenolic compounds					
Salidroside	Phenylpropanoid glycoside	C_14_H_20_O_7_	300	*Rhodiola rosea* (Crassulaceae)	[54]
Resveratrol *	Stilbene	C_14_H_12_O_3_	228	-	[55]
Curcumin *	Diarylheptanoid	C_21_H_20_O_6_	368	-	[56]
Hesperidin *	Flavanone glycoside	C_28_H_34_O_15_	610	-	[57]
Quercetin *	Flavone	C_15_H_10_O_7_	302	-	[58]
Baicalin	Flavone	C_21_H_18_O_11_	446	*Scutellaria baicalensis* (Lamiaceae)	[59]
Baicalein	Flavone	C_15_H_10_O_5_	270	*Scutellaria baicalensis* (Lamiaceae)	[60]
(-)-Epigallocatechin-3-gallate	Flavan	C_22_H_18_O_11_	458	*Camellia sinensis* (Theaceae)	[61]
Galangin	Flavonol	C_15_H_10_O_5_	270	*Alpinia officinarum* (Zingiberaceae)	[62]
Scutellarin *	Glycosyloxyflavone	C_21_H_18_O_12_	462	*-*	[63]
Astilbin	Flavanonol glucoside	C_21_H_22_O_11_	450	*Smilacis Glabra* (Smilacaceae)	[64]
Dihydroquercetin *	Flavanonol	C_15_H_12_O_7_	290	-	[65]
Shikonin *	Naphthoquinone	C_16_H_16_O_5_	288	-	[66]
Magnolol	Lignan	C_18_H_18_O_2_	266	*Magnolia officinalis* (Magnoliaceae)	[67]
Pterostilbene *	Stilbene	C_16_H_16_O_3_	256	-	[68]
Salvianolic acid	Phenolic acid	C_26_H_22_O_10_	494	*Salvia miltiorrhiza* (Lamiaceae)	[69]
Chlorogenic acid *	Phenolic acid	C_16_H_18_O_9_	354	*-*	[70]
Coumarins and coumarin derivatives					
Wedelolactone	Coumarin	C_16_H_10_O_7_	314	*Eclipta prostrata* (Asteraceae)	[71]
Osthole	Coumarin	C_15_H_16_O_3_	224	*Cnidium monnieri* (Apiaceae)	[72]
					[73]
Imperatorin	Psoralen	C_16_H_14_O_4_	270	*Cnidium monnieri* (Apiaceae)	[73]
Pd-Ia	Coumarin	C_21_H_22_O_7_	386	*Angelica decursiva* (Apiaceae)	[72]
Pd-II	Coumarin	C_24_H_26_O_7_	426	*Angelica decursiva* (Apiaceae)	[72]
Pd-III	Coumarin	C_24_H_28_O_7_	428	*Angelica decursiva* (Apiaceae)	[72]
		Other compounds					
Genipin	Iridoid	C_11_H_14_O_5_	226	*Gardenia jasminoides* (Rubiaceae)	[74]
α-Lipoic acid *	Organosulfur compound	C_8_H_14_O_2_S_2_	206	-	[75]
Allicin	Organosulfur compound	C_6_H_10_OS_2_	162	*Allium sativum* (Amaryllidaceae)	[76]
Protectin D1 *	Dihydroxydocosahexaenoic acid	C_22_H_32_O_4_	360	-	[77]
Salecan	Polysaccharide		2 × 10^6^ Da	*Agrobacterium* sp.ZX09 (Rhizobiaceae)	[78]
Fucoidan	Sulfated polysaccharide		13 kDa to 950 kDa	*Fucus vesiculosus* (Fucaceae)	[79]

* Purchased.

**Table 2:** Effect of the pretreatment with some natural products on Con A induced changes

**Compound Name**	**Measured Parameter**	**Results of Compound on Con A-Induced Change (Conc.)**	**Positive Control and/or Con A Model**	**Ref.**
Sophocarpine (SC)	ALT	398.02 U/L SC (30 mg/kg) 171.25 U/L SC (60 mg/kg)	628.12 U/L Bicyclol (100 mg/kg) 957.92 U/L Con A (15 mg/kg)	[10]
	AST	157.69 U/L SC (30 mg/kg) 84.46 U/L SC (60 mg/kg)	219.81 U/L Bicyclol (100 mg/kg) 344.98 U/L Con A (15 mg/kg)	
	TBIL	5.05 U/L SC (30 mg/kg) 3.21 U/L SC (60 mg/kg)	5.08 U/L Bicyclol (100 mg/kg) 9.99 U/L Con A (15 mg/kg)	
	% TUNEL-positive cells	8.43% SC (30 mg/kg) 4.33% SC (60 mg/kg)	6.23% Bicyclol (100 mg/kg) 13.53% Con A (15 mg/kg)	
	Relative MIP-1α expression	16.80-fold SC (30 mg/kg) 3.32-fold SC (60 mg/kg)	41.47-fold Con A (15 mg/kg)	
	Relative CXCL10 expression	57.05-fold SC (30 mg/kg) 18.56-fold SC (60 mg/kg)	123.72-fold Con A (15 mg/kg)	
	Relative ICAM-1 expression	20.69-fold SC (30 mg/kg) 3.35-fold SC (60 mg/kg)	43.44-fold Con A (15 mg/kg)	
	IFN-γ	2831.00 pg/mL SC (30 mg/kg) 2030.00 pg/mL SC (60 mg/kg)	3248.00 pg/mL Con A (15 mg/kg)	
	TNF-α	1009.78 pg/mL SC (30 mg/kg) 540.99 pg/mL SC (60 mg/kg)	998.35 pg/mL Con A (15 mg/kg)	
	% CD4^+^IFN-γ^+^	1.87% SC (30 mg/kg) 1.41% SC (60 mg/kg)	2.89% Con A (15 mg/kg)	
	Expression of T-bet	36.81 SC (30 mg/kg) 27.99 SC (60 mg/kg)	67.74 pg/mL Con A (15 mg/kg)	
	Activation of STAT1	75.72 SC (30 mg/kg) 8.21 SC (60 mg/kg)	205.49 pg/mL Con A (15 mg/kg)	
	Overexpression of SOCS1	2.21-fold SC (30 mg/kg) 3.83-fold SC (60 mg/kg)	1.76 pg/mL Con A (15 mg/kg)	
Demethyleneberberine (DMB)	ALT	↓ 91% DMB (30 mg/kg)	↑ 75-fold Con A (20 mg/kg)	[9]
	AST	↓ 85% DMB (30 mg/kg)	↑ 19-fold Con A (20 mg/kg)	
	LDH	↓ 48% DMB (30 mg/kg)	↑ 8-fold Con A (20 mg/kg)	
	MDA	↓ 29% DMB (7.5 mg/kg)	↑ 127% Con A (20 mg/kg)	
		↓ 40% DMB (15 mg/kg)		
		↓ 48% DMB (30 mg/kg)		
	ALB	↑ 16% DMB (15 mg/kg)	↓ 33% Con A (20 mg/kg)	
	GSH	↑ 45% DMB (7.5 mg/kg)	↓ 39% Con A (20 mg/kg)	
		↑ 61% DMB (15 mg/kg)		
		↑ 90% DMB (30 mg/kg)		
	TNF-α	↓ 64% DMB (15 mg/kg)	↑ 22-fold Con A (20 mg/kg)	
	IL-6	↓ 75% DMB (15 mg/kg)	↑ 42-fold Con A (20 mg/kg)	
	IL-1β	↓ 40% DMB (15 mg/kg)	↑ 21-fold Con A (20 mg/kg)	
	IFN-γ	↓ 85% DMB (15 mg/kg)	↑ 58-fold Con A (20 mg/kg)	
Fumigaclavine C (FCC)	ALT	3387 Karmen unit FCC (5 mg/kg)	650 Karmen unit Ciclosporin (10 mg/kg) 6288 Karmen unit Con A (18 mg/kg)	[33]
		1078 Karmen unit FCC (10 mg/kg)		
		447 Karmen unit FCC (20 mg/kg)		
	AST	4213 Karmen unit FCC (5 mg/kg)	1509 Karmen unit Ciclosporin (10 mg/kg) 8632 Karmen unit Con A (18 mg/kg)	
		1423 Karmen unit FCC (10 mg/kg)		
		921 Karmen unit FCC (20 mg/kg)		
	Hepatocyte necrosis *	1 (0–2) FCC (5 mg/kg)	0 (0–2) Ciclosporin (10 mg/kg) 2 (1–2) Con A (18 mg/kg)	
		0 (0–1) FCC (10 mg/kg)		
		0 (0–1) FCC (20 mg/kg)		
	Hepatocyte degeneration *	2 (1–3) FCC (5 mg/kg)	1 (0–3) Ciclosporin (10 mg/kg) 3 (1–3) Con A (18 mg/kg)	
		1 (0–2) FCC (10 mg/kg)		
		1 (0–1) FCC (20 mg/kg)		
	Inflammatory infiltration *	1 (1–2) FCC (5 mg/kg)	1 (1–2) Ciclosporin (10 mg/kg) 2 (1–3) Con A (18 mg/kg)	
		1 (1–2) FCC (10 mg/kg)		
		1 (0–2) FCC (20 mg/kg)		
	Kupffer cell hyperplasia *	1 (1–2) FCC (5 mg/kg)	2 (1–2) Ciclosporin (10 mg/kg) 1 (0–2) Con A (18 mg/kg)	
		1 (1–2) FCC (10 mg/kg)		
		1 (0–1) FCC (20 mg/kg)		
	Spleen weights	162.7 mg FCC (5 mg/kg)	124.4 mg Ciclosporin (10 mg/kg) 210.9 mg Con A (18 mg/kg)	
		121.4 mg FCC (10 mg/kg)		
		111.6 mg FCC (20 mg/kg)		
Halofuginone (HF)	Liver-to-bodyweight ratio (LBWR)	4.86 HF (10 ppm)	5.42 Con A (17.5mg/kg)	[34]
	ALT	59.29 IU/L HF (10 ppm)	73.37 IU/L Con A (17.5 mg/kg)	
	AST	193.02 IU/L HF (10 ppm)	274.64 IU/L Con A (17.5 mg/kg)	
	ALB	31.17 g/L HF (10 ppm)	27.58 g/L Con A (17.5 mg/kg)	
	Semi-quantitative scoring system (SSS) value	13 HF (10 ppm)	10 Con A (17.5 mg/kg)	
Soyalkaloid A (SAA)	Proliferation of Con A-activated lymphocytes	0.40 SAA (0.25 µM)	0.28 Cyclosporine A (1 µM) 0.43 Con A (1 µg/mL)	[37]
		0.36 SAA (0.5 µM)	0.28 Cyclosporine A (1 µM) 0.43 Con A (1 µg/mL)	
		0.32 SAA (1 µM)	0.28 Cyclosporine A (1 µM) 0.43 Con A (1 µg/mL)	
		0.29 SAA (2 µM)	0.28 Cyclosporine A (1 µM) 0.43 Con A (1 µg/mL)	
(5*R*)-5-Hydroxytriptolide (LLDT-8)	Survival rat	83% LLDT-8 (0.5 mg/kg)	40% Con A (30 mg/kg)	[41]
		86% LLDT-8 (1 mg/kg)		
		100% LLDT-8 (2 mg/kg)		
	ALT	952 U/L LLDT-8 (2 mg/kg)	2304 U/L Con A (30 mg/kg)	
	IFN-γ	1508 pg/mL LLDT-8 (2 mg/kg)	4375 pg/mL Con A (30 mg/kg)	
Magnesium isoglycyrrhizinate (MIG)	ALT	126.73 U/L MIG (50 mg/kg)	82.38 U/L Dexamethasone (2.5 mg/kg) 341.19 U/L Con A (20 mg/kg)	[44]
		156.78 U/L MIG (25 mg/kg)		
		212.40 U/L MIG (12.5 mg/kg)		
	AST	227.50 U/L MIG (50 mg/kg)	169.2 U/L Dexamethasone (2.5 mg/kg) 477.71 U/L Con A (20 mg/kg)	
		279.39 U/L MIG (25 mg/kg)		
		336.0 U/L MIG (12.5 mg/kg)		
	MDA	59.68 mmol/mg Prot MIG (50 mg/kg)	57.40 mmol/mg Prot Dexamethasone (2.5 mg/kg) 92.81 mmol/mg Prot Con A (20 mg/kg)	
		57.48 mmol/mg Prot MIG (25 mg/kg)		
		70.31 mmol/mg Prot MIG (12.5 mg/kg)		
	SOD	287.01 U/mg Prot MIG (50 mg/kg)	280.60 U/mg Prot Dexamethasone (2.5 mg/kg) 168.87 U/mg Prot Con A (20 mg/kg)	
		295.97 U/mg Prot MIG (25 mg/kg)		
		279.84 U/mg Prot MIG (12.5 mg/kg		
	TNF-α	348.69 pg/mL MIG (50 mg/kg)	183.44 pg/mL Dexamethasone (2.5 mg/kg) 498.65 pg/mL Con A (20 mg/kg)	
		301.77 pg/mL MIG (25 mg/kg)		
		311.40 pg/mL MIG (12.5 mg/kg)		
	IFN-γ	577.70 pg/mL MIG (50 mg/kg)	1786.73 pg/mL Dexamethasone (2.5 mg/kg) 182.33 pg/mL Con A (20 mg/kg)	
		466.53 pg/mL MIG (25 mg/kg)		
		792.51 pg/mL MIG (12.5 mg/kg)		
Cycloastragenol (CAG)	CD69^+^ expression	50.22% CAG (5.0 µM)	68.86% Con A (5 µg/mL)	[47]
		49.39% CAG (10 µM)		
		38.33% CAG (20 µM)		
	CD25^+^ expression	67.29 % CAG (5.0 µM)	90.45% Con A (5 µg/mL)	
		49.23% CAG (10 µM)		
		36.66% CAG (20 µM)		
	Con A-stimulated lymphocyte proliferation (PI)	1.35 CAG (5.0 µM)	1.43 Con A (5 µM)	
		1.29 CAG (10 µM)		
		1.07 CAG (20 µM)		
Eugenol	SOD	0.39 U/mg protein Eugenol (5 mg/kg)	0.25 U/mg protein Con A (12 mg/kg)	[8]
	CAT	44.5 nmol H_2_O_2_ utilized/mg protein Eugenol (5 mg/kg)	31.4 nmol H_2_O_2_ utilized/mg protein Con A (12 mg/kg)	
	GPx	6.41 nmol GSH utilized/mg protein Eugenol (5 mg/kg)	4.11 nmol GSH utilized/mg protein Con A (12 mg/kg)	
	GR	19 nmol NADPH oxidized/min/mg protein Eugenol (5 mg/kg)	11 nmol NADPH oxidized/min/mg protein Con A (12 mg/kg)	
	G6PD	1.46 nmol inorganic phosphorus liberated/min/mg protein Eugenol (5 mg/kg)	1.14 nmol inorganic phosphorus liberated/min/mg protein Con A (12 mg/kg)	
Paeoniflorin	% CD4^+^ infiltration	6.37% Paeoniflorin (50 mg/kg)	17.4% Con A (15 mg/kg)	[38]
	% CD8^+^ infiltration	6.56% Paeoniflorin (50 mg/kg)	13.3% Con A (15 mg/kg)	
	Amount NKT infiltration	8.89 × 10^5^ Paeoniflorin (50 mg/kg)	14.56 × 10^5^ Con A (15 mg/kg)	
Resveratrol (RSV)	ALT	351.98 IU/L RSV (30 mg/kg)	853.13 IU/L Con A (15 mg/kg)	[80]
	AST	295.86 IU/L RSV (30 mg/kg)	753.98 IU/L Con A (15 mg/kg)	
	TNF-α	159.73 pg/mL RSV (30 mg/kg)	314.98 pg/mL Con A (15 mg/kg)	
	IL-6	215.87 pg/mL RSV (30 mg/kg)	513.47 pg/mL Con A (15 mg/kg)	
	IFN-γ	137.75 pg/mL RSV (30 mg/kg)	285.95 pg/mL Con A (15 mg/kg)	
	MCP-1	203.13 pg/mL RSV (30 mg/kg)	837.98 pg/mL Con A (15 mg/kg)	
	ALT	253.73 IU/L RSV (10 mg/kg)	389.40 IU/L Con A (20 mg/kg)	[55]
		239.40 IU/L RSV (20 mg/kg)		
		205.52 IU/L RSV (30 mg/kg)		
	AST	250.49 IU/L RSV (10 mg/kg)	450.74 IU/L Con A (20 mg/kg)	
		259.89 IU/L RSV (20 mg/kg)		
		165.36 IU/L RSV (30 mg/kg)		
	Knodell scores	4.67 RSV (10 mg/kg)	5.51 Con A (20 mg/kg)	
		3.33 RSV (20 mg/kg)		
		3.0 RSV (30 mg/kg)		
Curcumin (CM)	ALT 2 h	123.8 IU/L CM (200 mg/kg)	155.0 IU/L Con A (20 mg/kg)	[11]
	ALT 8 h	629.2 IU/L CM (200 mg/kg)	833.7 IU/L Con A (20 mg/kg)	
	ALT 24 h	263.0 IU/L CM (200 mg/kg)	435.7 IU/L Con A (20 mg/kg)	
	AST 2 h	475.2 IU/L CM (200 mg/kg)	469.8 IU/L Con A (20 mg/kg)	
	AST 8 h	881.3 IU/L CM (200 mg/kg)	1143.8 IU/L Con A (20 mg/kg)	
	AST 24 h	377.8 IU/L CM (200 mg/kg)	629.3 IU/L Con A (20 mg/kg)	
Hesperidin (HDN)	T-cell activation ratio in vivo (after 6 h)	70.05 HDN (1000 mg/kg)	90.21 Con A (15 mg/kg)	[57]
	T-cell activation ratio in vitro (after 12 h)	64.59 HDN (1000 mg/kg)	83.39 Con A (15 mg/kg)	
Scutellarin	ALT	410.7 U/L Scutellarin (50 mg/kg)	550.0 U/L Con A (25 mg/kg)	[63]
		402.2 U/L Scutellarin (100 mg/kg)		
	AST	224.4 U/L Scutellarin (50 mg/kg)	448.0 U/L Con A (25 mg/kg)	
		229.9 U/L Scutellarin (100 mg/kg)		
	Histological grade of liver injury	1.6 Scutellarin (100 mg/kg)	2.6 Con A (25 mg/kg)	
	TNF-*α*	1082.6 pg/mL Scutellarin (50 mg/kg)	1067.9 pg/mL Con A (25 mg/kg)	
		477.1 pg/mL Scutellarin (100 mg/kg)		
	NO_2_^-^/NO_3_^-^ levels	21.1 µM/L Scutellarin (50 mg/kg)	24.8 µM/L Con A (25 mg/kg)	
		13.5 µM/L Scutellarin (100 mg/kg)		
	iNOS/β-actin	1.48 Scutellarin (50 mg/kg)	2.29 Con A (25 mg/kg)	
		0.95 Scutellarin (100 mg/kg)		
	IFN-γ/β-actin	1.94 Scutellarin (50 mg/kg)	2.50 Con A (25 mg/kg)	
		1.85 Scutellarin (100 mg/kg)		
	Fas/β-actin	2.05 Scutellarin (50 mg/kg)	2.82 Con A (25 mg/kg)	
		1.81 Scutellarin (100 mg/kg)		
	TNF-α/β-actin	2.03 Scutellarin (50 mg/kg)	3.00 Con A (25 mg/kg)	
		1.13 scutellarin (100 mg/kg)		
Astilbin	Hepatocyte necrosis	0.75 Scutellarin (20 mg/kg)	0 Ciclosporin (10 mg/kg) 1.13 Con A (20 mg/kg)	[64]
		0.63 Astilbin (40 mg/kg)		
	Hepatocyte degeneration	1.5 Astilbin (20 mg/kg)	0.25 Ciclosporin (10 mg/kg) 2.25 Con A (20 mg/kg)	
		0.88 Astilbin (40 mg/kg)		
	Inflammatory infiltration	1.13 Astilbin (20 mg/kg)	0.88 Ciclosporin (10 mg/kg) 1.5 Con A (20 mg/kg)	
		0.75 Astilbin (40 mg/kg)		
	Kupffer cell hyperplasia	1 Astilbin (20 mg/kg)	0.75 Ciclosporin (10 mg/kg) 1.38 Con A (20 mg/kg)	
		0.75 Astilbin (40 mg/kg)		
Pterostilbene (PTE)	ALT	390 U/L PTE (10 mg/kg)	1400 U/L Con A (10 mg/kg)	[68]
		130 U/L PTE (40 mg/kg)		
	AST	500 U/L PTE (10 mg/kg)	1700 U/L Con A (10 mg/kg)	
		230 U/L PTE (40 mg/kg)		
	TUNEL positive cells	≈14% PTE (10 mg/kg)	≈41% Con A (10 mg/kg)	
		≈6% PTE (40 mg/kg)		
	Ki67 positive area	≈10% PTE (10 mg/kg)	≈1% Con A (10 mg/kg)	
		≈13% PTE (40 mg/kg)		
	mRNA IFN-γ level	↓≈2.7% PTE (10 mg/kg) ↓≈3.3% PTE (40 mg/kg)	-	
	mRNA TNF-α level	↓≈3.1 PTE (10 mg/kg) ↓≈4.5% PTE (40 mg/kg)	-	
	TF	↓≈19% PTE (10 mg/kg)	↑≈43% Con A (10 mg/kg)	
		↓≈6% PTE (40 mg/kg)		
	Fibrin	↓≈3.8 PTE (10 mg/kg)	-	
		↓≈6.7% PTE (40 mg/kg)		
	F4/80 staining	↓≈11% PTE (10 mg/kg)	↑≈42% Con A (10 mg/kg)	
		↓≈5% PTE (40 mg/kg)		
Ursodeoxycholic acid (UDCA)	UDCA	38.5 nmol/g liver UDCA (50 mg/kg)	2.4 nmol/g liver Con A (20 mg/kg)	[52]
		87.8 nmol/g liver UDCA (150 mg/kg)		
	α-MCA	11.1 nmol/g liver UDCA (50 mg/kg)	13.1 nmol/g liver Con A (20 mg/kg)	
		18.7 nmol/g liver UDCA (150 mg/kg)		
	β-MCA	58.7 nmol/g liver UDCA (50 mg/kg)	72.3 nmol/g liver Con A (20 mg/kg)	
		49.7 nmol/g liver UDCA (150 mg/kg)		
	HDCA	<1 nmol/g liver UDCA (50 mg/kg)	<1 nmol/g liver Con A (20 mg/kg)	
		<1 nmol/g liver UDCA (150 mg/kg)		
	CA	107.8 nmol/g liver UDCA (50 mg/kg)	141.1 nmol/g liver Con A (20 mg/kg)	
		105.5 nmol/g liver UDCA (150 mg/kg)		
	CDCA	<1 nmol/g liver UDCA (50 mg/kg)	<1 nmol/g liver Con A (20 mg/kg)	
		<1 nmol/g liver UDCA (150 mg/kg)		
	DCA	6.5 nmol/g liver UDCA (50 mg/kg)	10.0 nmol/g liver Con A (20 mg/kg)	
		12.4 nmol/g liver UDCA (150 mg/kg)		
	LCA	<1 nmol/g liver UDCA (50 mg/kg)	<1 nmol/g liver Con A (20 mg/kg)	
		<1 nmol/g liver UDCA (150 mg/kg)		
	Total bile acids	223.6 nmol/g liver UDCA (50 mg/kg)	239.6 nmol/g liver Con A (20 mg/kg)	
		275.7 nmol/g liver UDCA (150 mg/kg)		

* The histological changes were read on a scale of 0–3 (0: no change; 1: mild; 2: moderate; 3: severe).

**Table 3:** Plant extracts, faction, and/or herbal formulation effects on con A-induced liver injury

Name of Plant (Organ, Family)//Preparation	Tested Extract/Fraction (Major Constituents)	Extract/Fraction/Con A (Conc.)	Pharmacological Outcomes/Effects	Reference
Yin-Chen-Hao (YCH) prescription in China inchinkoto (ICKT) and Inchinko-To (TJ-135) in Japan *Artemisia capillaris* (Inchinko) (Herb, Asteraceae) *Gardenia jasminoides* (Sanshishi) (Fruit, Rubiaceae) *Rheum palmatum* (Daio) (Rhizome, Polygonaceae)	Aqueous extract (ICKTD) (capillarisin, genipin, and 6,7-dimethylesculetin)	ICKTD (500 mg/10 mL/kg) ICKTD (1000 mg/10 mL/kg) ICKTD (2000 mg/10 mL/kg) Con A (20 mg/kg)	ICKTD ↓ ALT, AST, and IFN-γ concentrations. ICKTD ↓ production of IFN-γ in con A-stimulated splenocyte culture in vitro. ICKTD suppressed IL-1β, IL-6, and IL-12p70 synthesis. ICKT ↓ nitrite release from IFN-γ-stimulated macrophages.	[83]
	Aqueous extract (decoction) (YCHD) (0.32% scoparone, 0.96% geniposide and 0.25% rhein)	YCH (150 mg/kg) YCH (300 mg/kg) YCH (600 mg/kg) Con A (22 mg/kg)	YCHD ↓ elevation in ALT, AST, and LDH activity. YCHD ↓ liver DNA fragmentation and caspase-3 levels. YCHD inhibited in vitro and in vivo TNF-α production. YCHD blocked the activation of NF-κB. YCHD effect depended on ↓TNF-α production via inhibition of NF-κB activation.	[84]
			
	Hot-water extract (TJ-135)	TJ-135 (500 mg/10 mL/kg) TJ-135 (1000 mg/10 mL/kg) TJ-135 (2000 mg/10 mL/kg) Con A (14 mg/kg)	TJ-135 significantly ↓serum AST, ALT, and LDH levels. TJ-135 suppressed sub-massive hepatic necrosis accompanying inflammatory cell infiltration. TJ-135 ↓ serum levels of IL-12, IFN-γ, and IL-12. TJ-135 ↑ IL-10 serum and intrasplenic levels. TJ-135 inhibited the production of inflammatory cytokine and enhanced the production of anti-inflammatory cytokines.	[85]
*Angelica sinensis* (Roots, Apiaceae)	Polysaccharide (arabinose, glucose, and galactose with molar ratio 1:2.5:7.5) (ASP)	ASP (1.6 mg/kg) ASP (6 mg/kg) Con A (15 mg/kg)	ASP attenuated Con A-induced liver injury through its anti-inflammatory and anti-oxidant actions. ASP ↓ ALT and AST levels. ASP ↓ pro-inflammatory cytokines: TNF-α, IFN-γ, IL-2 and IL-6 levels. ASP alleviated oxidative stress by ↓ MDA and ROS levels and ↑ SOD activity. ASP ↓ caspase-3-dependent apoptosis by Caspase-8 and JNK-mediated pathway. ASP inhibited IL-6/STAT3 and NF-κB signaling pathways activation.	[86]
*Malus domestica* (Fruits, Rosaceae)	Polyphenols (AP) (procyanidins, quercetin, (-)-epicatechin, (+)-catechin, phloretin, phloridzin, and chlorogenic acid)	AP (200 mg/kg) AP (400 mg/kg) AP (800 mg/kg) Con A (200 mg/kg)	AP ↓ ALT, AST, TP and Alb levels and A/G ratio. AP ↓ TNF-α and IFN-γ, ↓ NO content serum levels. AP inhibited oxidative DNA single-strand breaks. AP improved the abnormalities of MDA content, SOD activity, and GSH level. AP exerted a protective effect through activation of the antioxidant system and suppression of pro-inflammatory cytokines.	[87]
*Rubus occidentalis* (Fruit, Rosaceae)	Ethanol/H_2_O soluble extract (BRB-A) Hexane extract (BRB-B) Ethanol/H_2_O insoluble extract (BRB-C)	BRB-A (1.6 mg/kg) BRB-B (1.6 mg/kg) BRB-C (1.6 mg/kg) Con A (30 mg/kg)	BRB-A and BRB-C inhibited Con A induced liver injury. The BRB-A and BRB-C ↓ lipid peroxidation and NDA oxidative damage. BRB-A and BRB-C ↑ MnSOD activity but not the Cu/ZnSOD. BRB extracts protective action resulted from their antioxidant action.	[88]
*Citrus grandis* (Fruit, Rutaceae)	EtOH extract (ECGO)	ECGO (3.9, 7.8, 15.6, 31.3, 62.5,125, 250, 500, and 1000 µg/mL) Con A (10 µg/mL)	ECGO significantly ↓ CD44/CD62L^+^ T cell population and ↓ production of IL-2, IFN-γ and IL-4 in Con A-stimulated splenocytes.	[89]
*Ligustrum lucidum* (Fruit, Oleaceae) (FLL)	Aqueous fraction (FLL-Aq) Butanol fraction (FLL-Bu) Chloroform fraction (FLL-Ch) Petroleum ether fraction (FLL-Pe)	FLL-Aq (2 g/kg) FLL-Bu (1 g/kg) FLL-Ch (1 g/kg) FLL-Pe (1 g/kg) Con A (1, 2, 4 μg/mL)	FLL-Pe produced an immunostimulatory effect, as evidenced by the significant enhancement of the Con A-stimulated mitogenic response of splenocytes in in vitro culture. FLL-Bu and FLL-Aq inhibited the in vitro Con A-stimulated splenocyte proliferation. FLL-Pe or FLL-Ch in ex vivo experiments, enhanced the extent of Con A-stimulated splenocyte proliferation. FLL-Pe and FLL-Ch produced immuno-stimulatory action under ex vivo assay conditions, but only FLL-Pe produced a similar effect under in vitro conditions.	[90]
Propolis (resinous natural product from honeybees)	phenolic acids or their esters, flavonoids, terpenes, fatty acids, stilbenes, and β-steroids	Propolis (300 mg/kg) Con A (12 mg/kg)	Propolis significantly ↑ Alb level and ↓ ALT, AST, and total bilirubin levels. Propolis attenuated lipid peroxidation and ↑ GSH, SOD, and CAT activities in liver tissue. Propolis ↓ serum levels of TNF-α and IL-6. Propolis ↓ TGF-β activation and Smad phosphorylation.	[91]
*Rabdosia amethystoides* (Root, Lamiaceae)	EtOH extract (ERA)	ERA (50 mg/kg) ERA (100 mg/kg) ERA (150 mg/kg) Con A (15 mg/mL/kg)	ERA significantly ↓ the elevated ALT and AST levels and liver necrosis in Con A-induced hepatitis. ERA significantly ↓ MPO and MDA levels and ↑ SOD level in the liver tissue. ERA significantly ↓ the expression level of TLR4 mRNA or protein in liver tissues. ERA attenuated the activation of the NF-kB pathway by inhibiting IkBα kinase and p65 phosphorylation. ERA effect may be mediated by the downregulation of TLR4 expression and inhibition of NF-kB activation.	[92]
*Zea mays* (Seeds, Poaceae)	Selenium-bio-fortified Peptides (SeCPs)	SeCPs (200 mg/kg) Con A (15 mg/kg)	SeCPs significantly ↓ AST, ALT, TNF-α, IFN-γ contents in serum, and MDA contents in liver. SeCPs significantly ↑ SOD and GPx activities. SeCPs effect was related to its antioxidant capacity, reduction of lipid peroxidation, and inhibiting the release of immune factors (TNF-α and IFN-γ)	[93]
*Tetrastigma hemsleyanum* (Roots, Vitaceae)	Total flavonoids (TFT)	TFT (1 mg/kg) TFT (2 mg/kg) TFT (4 mg/kg) Con A (20 mg/kg)	TFT significantly ↓ ALT and AST serum levels and attenuated histopathological alterations in Con A-induced liver injury. TFT ↓ increased serum levels of IL-17 and IL-6, Th17 cells proportions in spleen and the expression of RORγt in hepatic tissues. TFT enhanced % of Treg cells in spleen, the expression of Foxp3 in hepatic tissues and levels TGF-β1 and IL-10 in serum.	[94]
*Tupistra chinensis* (Rhizomes, Asparagaceae)	EtOH extract (TCE) (saponins)	TCE (75 mg/kg) TCE (150 mg/kg) TCE (300 mg/kg) Con A (15 mg/kg)	TCE significantly ↓ ALT, AST, and LDH serum levels. TCE ↓ IFN-γ and TNF-α levels. TCE suppressed MAPK and NF-κB-signaling in liver. TCE blocked STAT1/NF-κB-signaling and induced apoptosis in activated T cells.	[95]
Yiguanjian decoction (YD) *Glehnia littoralis* (Roots, Apiaceae) *Ophiopogon japonicus* (Roots, Asparagaceae) *Angelica sinensis* (Roots, Apiaceae) *Rehmannia glutinosa* (Roots, Scrophulariaceae) *Lycium barbarum* (Fruits, Solanaceae) *Melia toosendan* (Fruits, Meliaceae)	Aqueous extract	YD (0.13 g/mL) YD (0.26 g/mL) YD (0.52 g/mL) Con A (2 mg/mL)	YD improved the degree of liver inflammation and fibrosis in the liver of chronic hepatitis mice. YD ↓ liver cell DNA damage. YD ↓ protein expression of TNF-α in liver tissue. YD ↑ mRNA expression of Bax and MTH1.	[96]
Jiang-Xian HuGan (JXHG) herbal formula *Corbicula fluminea* (Clams, Cyrenidae) *Curcuma longa* (Rhizomes, Zingiberaceae) *Ligustrum lucidum* (Leaves, Oleaceae) *Eclipta prostrata* (Herb, Asteraceae) *Paeonia lactiflora* (Roots, Paeoniaceae)	EtOH extract	JXHG (700 mg/kg) JXHG (1400 mg/kg) Con A (20 mg/kg)	JXHG ↓ serum levels of ALT and AST, and ↓ hepatocyte apoptosis and mortality. JXHG significantly ↓ the serum levels of IFN-γ, TNF-α, IL-4, and IL-6. JXHG ↓ the mRNA expression of IL-6 and IFN-γ and ↑ IL-10, SOD1, GSR, and GPX2 mRNA in the liver tissues. JXHG dramatically suppressed NF-κB p65 phosphorylation, ↑ Nrf2 expression, and ↓ expression ratio of cleaved caspase-3/caspase-3 in liver tissues.	[97]

## Data Availability

No new data were created or analyzed in this study. Data sharing is not applicable to this article.

## Abbreviations

A/G ratio: albumin/globulin; ACC: acetyl coenzyme-A carboxylase; AIH: autoimmune hepatitis; AKT: Phospho-protein kinase B; ALB: albumin; ALP: alkaline phosphatase; ALT: alanine transaminase; AMPK: adenosine 5′-monophosphate (AMP)-activated protein kinase; AST: aspartate transaminase; Bax: Bcl-2 associated X protein; Bcl-2: B-cell lymphoma 2; CA: cholic acid; CAG: cycloastragenol; CAT: catalase; CBA: cytometric bead array; CCK8: cell counting kit-8; CCL5: C-C motif chemokine ligand 5; CCR1: C-C chemokine receptor 1; CCR5: C-C chemokine receptor 5; CDCA: chenodeoxycholic acid; CIH: concanavalin induced hepatitis; Con A: concanavalin A; CTLs: cytotoxic T lymphocytes; CX3CL1: chemokine (C-X3-C motif) ligand 1; CX3CR1: chemokine (C-X3-C motif) receptor 1; CXCL10: C-X-C motif chemokine 10; CXCL2: C-X-C motif chemokine 2; CXCR3: C-X-C chemokine receptor 3; Dbil: direct bilirubin; DCA: deoxycholic acid; G6PDH: glucose 6-phosphate dehydrogenase; Gli-1: glioblastoma-1; GM-CSF: granulocyte macrophage-colony stimulating factor; GPx: glutathione peroxidase; GR: glutathione reductase; GSH/GSSG: reduced/oxidized glutathione; GSH: glutathione; GST: glutathione S-transferase; HA: hyaluronic acid; HBsAg: hepatitis B surface antigen; HDCA: hyodeoxycholic acid; HMGB1: high-mobility group box 1; HO-1: heme-oxygenase-1; HSCs: hepatic stellate cells; ICAM-1: intercellular adhesion molecule-1; IFN-γ: interferon-gamma; IFN-γ-IP-10: IFN-γ-inducible protein-10; IHLs: intrahepatic leukocytes; IL-17: interleukin-17; IL-1β: interleukin-1β; IL-2: interleukin-2; IL-25: interleukin-25; IL-6: interleukin-6; iNOS: inducible nitric oxide synthase; IRF-1: interferon regulatory factor-1; I-TAC: IFN-inducible T cell-α chemoattractant; IκBα: inhibitory kappa B alpha; JAK2: Janus kinase 2; KCs: kupffer cells; LC3: microtubule-associated protein 1 light chain 3; LCA: lithocholic acid; LDH: lactate dehydrogenase; LLDT-8: (5R)-5-hydroxytriptolide; LPS: lipopolysaccharide; mAbs: monoclonal antibodies; MAPKs: mitogen-activated protein kinases; MCP-1: monocyte chemoattractant protein-1; MDA: malondialdehyde; MDSCs: myeloid-derived suppressor cells; Mig: monokine induced by IFN-γ; MIP-1α: macrophage inflammatory protein-1α; MIP-1β: macrophage inflammatory protein-1β; MIP-2: macrophage inflammatory protein-2; MLA: methyllycaconitine; MNCs: mononuclear cells; MnSOD: manganese superoxide dismutase; MPO: myeloperoxidase; MTH1: mutT homolog 1; NFAT: nuclear factor of activated T-cells; NF-κB: nuclear factor κB; NKT: natural killer T; NLRP3: pyrin domain containing 3; NP: neopterin; Nrf2: nuclear factor erythroid 2-related factor2; OPN: osteopontin; p-Cdk2: p-cyclin-dependent kinase 2; PCIII: procollagen III; PCNA: proliferating cell nuclear antigen; PD: platycodin D; PD1: protectin D1; PD2: platycodin D2; PF: paeoniflorin; PHAs: phytohemagglutinins; PI: proliferation index; PI3K: phosphatidylinositol 3 kinase; PKCθ: protein kinase Cθ; Pris: pristimerin; PSA: periplocoside A; Ptc: Patched; RANTES: regulated upon activation, normal T cell expressed, and secreted; ROS: reactive oxygen species; Shh: Sonic hedgehog; SOCS: suppressors of cytokine signaling; SOCS1: suppressor of cytokine signaling 1; SOD: superoxide dismutase; STA: stephanthraniline A; STAT1: signal transducer and activator of transcription 1; STAT3: signal transducer and activator of transcription 3; TBARS: thiobarbituric acid reactive substances; TBIL: total bilirubin; TBil: total bilirubin; TCR: T cell-receptor; TF: tissue factor; TGF-β1: transforming growth factor-β1; Th: T helper; Th1 cell: T-helper type 1 cell; Th17 cell: T-helper type 17 cell; Th2 cell: T-helper type 2 cell; TIMP2: tissue inhibitors of metalloproteinase 2; TLR: Toll-like receptor; TLR2: T toll-like receptor 2; TLR4: T toll-like receptor 4; TLR9: T toll-like receptor 9; TNF-α: tumor necrosis factor α; Treg: T regulatory cells; TUNEL: terminal deoxynucleotidyl transferase (TdT)-mediated dUTP-biotin nick end labeling; UDCA: ursodeoxycholic acid; VCAM-1: vascular cell adhesion molecule-1; ZAA: zhankuic acid A; α7-nAChR: α7-nicotinic acetylcholine receptors; α-GalCer: α-galactosylceramide; α-MCA: α-muricholic acid; α-SMA: alpha-smooth muscle actin; βcl-xL: β-cell lymphoma-extra-large; β-MCA:β-muricholic acid.
